# DGKα and ζ deficiency causes regulatory T-cell dysregulation, destabilization, and conversion to pathogenic T-follicular helper cells to trigger IgG1-predominant autoimmunity

**DOI:** 10.7554/eLife.105212

**Published:** 2026-09-18

**Authors:** Lei Li, Hongxiang Huang, Hongxia Wang, Yun Pan, Huishan Tao, Shimeng Zhang, Peer Karmaus, Michael B Fessler, John Sleasman, Xiao-Ping Zhong

**Affiliations:** 1 Department of Pediatrics-Allergy and Immunology, Duke University Medical Center Durham United States; 2 Immunity, Inflammation and Disease Laboratory, National Institute of Environmental Health Sciences, National Institutes of Health, Research Triangle Park Durham United States; 3 Department of Immunology, Duke Cancer Institute, Duke University Medical Center Durham United States; 4 Hematologic Malignancies and Cellular Therapies Program, Duke Cancer Institute, Duke University Medical Center Durham United States; Universidade Federal de Minas Gerais Brazil; The University of Tokyo Japan

**Keywords:** autoimmune response/disease, lymphocyte subsets, regulatory T cells, Mouse

## Abstract

Regulatory T cells (Tregs) actively engage in immune suppression to prevent autoimmune diseases, but also inhibit anti-tumor immunity. Although Tregs express a TCR repertoire with relatively high affinities to self, they are normally quite stable, and their inflammatory programs are intrinsically suppressed. We report here that diacylglycerol kinases (DGK) α and ζ are crucial for homeostasis, suppression of proinflammatory programs, and stability of Tregs, and for enforcing their dependence on CD28 costimulatory signal. Treg-specific deficiency of both DGKα and ζ derails signaling, metabolic, and transcriptional programs in Tregs to cause dysregulated phenotypic and functional properties and to unleash conversion to pathogenic exTregs, especially exTreg-T follicular helper (Tfh) 2 cells, leading to uncontrolled effector T cell differentiation, deregulated germinal center B-cell responses, and IgG1/IgE predominant antibodies/autoantibodies, and multiorgan autoimmune diseases. Our data not only illustrate the crucial roles of DGKs in Tregs to maintain self-tolerance, but also unveil a Treg-to-self-reactive-pathogenic-exTreg-Tfh-cell program that is suppressed by DGKs and that could exert broad pathogenic roles in autoimmune diseases if unchecked.

## Introduction

Regulatory T cells (Tregs) are crucial for maintaining self-tolerance to prevent autoimmune diseases (Sakaguchi et al., 2008; Li and Rudensky, 2016; Bluestone and Tang, 2018). The Treg-specific transcription factor (TF) Foxp3 is crucial for Treg generation, identity, and function (Hori et al., 2003; Fontenot et al., 2003). Natural Tregs are generated in the thymus, express TCRs with relatively high affinities to self-antigens, and display a certain degree of self-reactivity. However, Tregs express suppressive cytokines but not IL-2 and other proinflammatory cytokines associated with T helper cells (Takahashi et al., 1998). Foxp3 and Treg-specific developmentally established epigenetic landscapes suppress overt proinflammatory responses in Tregs (Samstein et al., 2012; Bettini et al., 2012; Zheng et al., 2010). Tregs can be pathogenic for autoimmune diseases if they lose suppressive functions, gain proinflammatory functions, and/or become unstable and convert to exTregs because of their self-reactivity (Hori, 2014; Sakaguchi et al., 2013; Zhou et al., 2009; Bailey-Bucktrout et al., 2013). However, mechanisms that control Treg stability and function are still not fully understood.

Autoantibodies are hallmarks for many autoimmune diseases. GC is a major site of Ig class-switch and antibody affinity maturation and, as such, are essential for protective humoral immune response and, unfortunately, development of autoantibodies (DeFranco, 2016; Crotty, 2015). Tfh cells promote GC B-cell proliferation and survival, Ig-class switch and affinity maturation, and memory B-cell and long-lived plasma cell formation (Crotty, 2015). However, deregulated Tfh-cells can trigger abnormal GC B and memory B-cell responses, leading to augmented autoantibody production (Arkatkar et al., 2017; Choi et al., 2016). T follicular regulatory (Tfr) cells, a specialized Treg sublineage expressing CXCR5, PD-1, and the TF Bcl6, suppress GC responses including autoantibody responses (Sage and Sharpe, 2016; Fu et al., 2018). Abnormal Tfh and Tfr-cells have been associated with or are the causal factors of autoimmune diseases in humans and animals (Choi et al., 2016; Fu et al., 2018; Jacquemin et al., 2015). However, the origins of pathogenic self-reactive Tfh-cells in autoimmune diseases are elusive.

TCR signal participates in Foxp3 induction, Treg-specific CpG hypomethylation formation, and Treg maintenance (Delpoux et al., 2014; Levine et al., 2014). A critical event after TCR engagement is PLCγ1-mediated generation of two important second messengers, diacylglycerol (DAG) and inositol tris-phosphates. DAG associates with and activates multiple effector molecules such as RasGRP1 and PKCθ, leading to activation of the Ras-Erk1/2 and IKKα/β/γ-NFκB pathways as well as PI3K/Akt-mTOR signaling (Gorentla et al., 2011). DAG can be controlled by DGKs, a family of 10 isoforms phosphorylating DAG to produce phosphatidic acid (Chen et al., 2016; Mérida et al., 2019). DGKα and DGKζ, the major isoforms expressed in T-cells, regulate DAG-mediated RasGRP1-Ras-Erk1/2, PKCθ-IKK-NFκB, and PI3K/Akt-mTOR pathways in T cells to control their development, activation, anergy, survival, effector function, and antimicrobial and antitumor immunity, as well as *i*NKT and MAIT-cell development (Gorentla et al., 2011; Zhong et al., 2003; Zha et al., 2006; Guo et al., 2008; Olenchock et al., 2006; Baldanzi et al., 2011; Yang et al., 2016b; Ruffo et al., 2016; Jung et al., 2018; Riese et al., 2013; Shen et al., 2011; Pan et al., 2020; Yang et al., 2019). Although deficiency of DGKζ alone facilitated Treg development (Schmidt et al., 2013; Joshi et al., 2013), whether DGKα and ζ may function synergistically in Tregs, and their true in vivo importance in Tregs for self-tolerance, have remained unclear.

We report here that DGKα and ζ synergistically ensure proper signaling, metabolic, and transcriptional programs in Tregs to ensure their normal homeostasis, stability, and lack of inflammatory property, and to enforce their dependence on CD28 costimulatory signal. DGKα and ζ double-deficiencies (DKO) in Tregs unleash their proinflammatory programs, alleviate CD28-dependence for their development and homeostasis, and facilitate their conversion to pathogenic exTregs, especially exTreg-Tfh2-cells, causing multiorgan autoimmune diseases, deregulated GC B-cell responses, and IgG1/IgE predominant autoantibodies, and lupus-like diseases.

## Results

### Treg-specific DGKα and ζ double deficiency causes multiorgan autoimmune diseases

**Figure 1.**
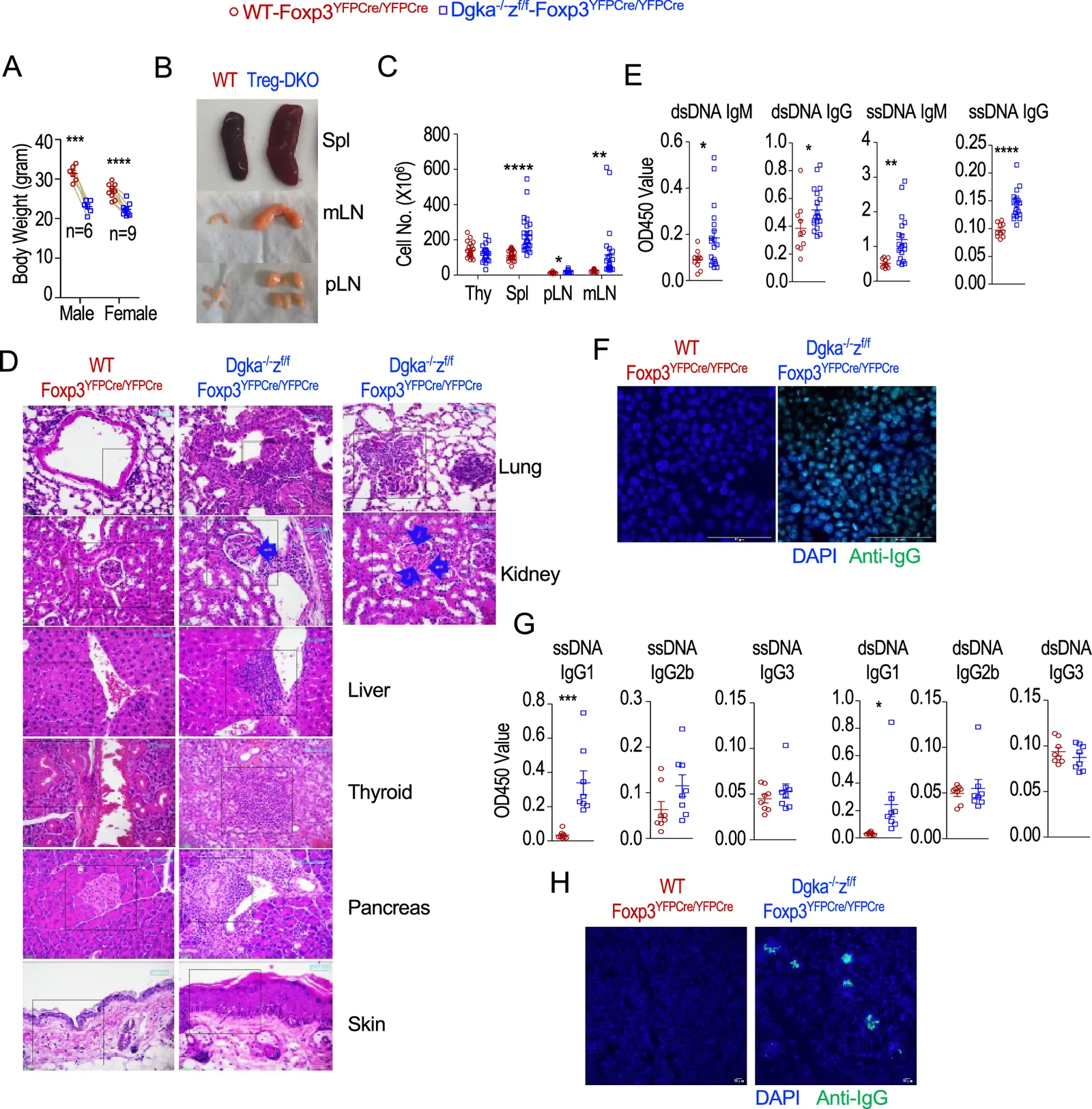
Autoimmune diseases in Treg-αζDKO mice. Female *Dgka^-/-^z^f/f^-Foxp3^YFPCre/YFPCre^* or male *Dgka^-/-^z^f/f^-Foxp3^YFPCre^* and WT control mice were analyzed. (**A**) Body weights of 5- to 9-month-old mice. (**B**) Representative pictures of indicated organs in a pair of 6-month-old mice. (**C**) Total cell numbers in the indicated organs in 2- to 9-month-old mice. (**D**) Representative H&E staining of paraffin thin-sections of indicated organs. (**E**) Seral autoantibody titers in 7-month-old mice. (**F**) Representative images of detection of seral antinuclear antibodies against fixed HEp-2 cells. (**G**) Seral autoantibody IgG subtypes. (**H**) Detection of IgG deposition in cryo-sections of kidneys with fluorescence confocal microscopy. Data shown are representative of or pooled from at least six experiments. Each circle or square represents one mouse of the indicated genotypes. Each line connecting the circle and square represents one pair of age- and sex-matched mice examined in one experiment. *, p<0.05; **, p<0.01; ***, p<0.001; ****, p<0.0001 determined by two-tailed pair-wise (**A**, data with lines connecting WT and αζDKO mice) or unpaired Student's *t* test.

To determine the role of DGKα and ζ in Tregs for self-tolerance, we analyzed *Dgka^-/-^z^f/f^-Foxp3^YFPCre/YFPCre^* Treg-specific DGKαζDKO (DKO-Cre or Treg-αζDKO) and control WT*-Foxp3^YFPCre/YFPCre^* (WT-Cre) mice. Treg-αζDKO mice had weight losses (Figure 1) and developed lymphoproliferative disorders and multiorgan autoimmune diseases, manifested by enlarged spleen and lymph nodes (LNs) with increased total cellularity (Figure 1B and C), mononuclear cell infiltration in the liver, lung, kidney, thyroid gland, and pancreas, significant thickening of the epidermis of skin (Figure 1D, Figure 1—figure supplement 1), and elevated anti-double strand DNA (dsDNA), -single strand DNA (ssDNA), and -nuclear autoantibodies (Figure 1E and F). Elevated autoantibodies were mainly caused by increased IgG1 but not IgG2b or IgG3 (Figure 1G), suggesting enhanced type-2 autoimmunity. In the kidney, thickened glomerular basal membrane (arrows in Figure 1D) and IgG deposition in the glomeruli could be observed (Figure 1H). Although DGKα and ζ can function individually to promote T cell anergy and enhance T cell activation in certain experimental settings, neither DGKζ nor DGKα deficiency caused obvious lymphoproliferative or autoimmune diseases (Figure 1—figure supplement 2). Thus, DGKα and ζ function synergistically in Tregs to ensure self-tolerance.

**Figure 1—figure supplement 1.**
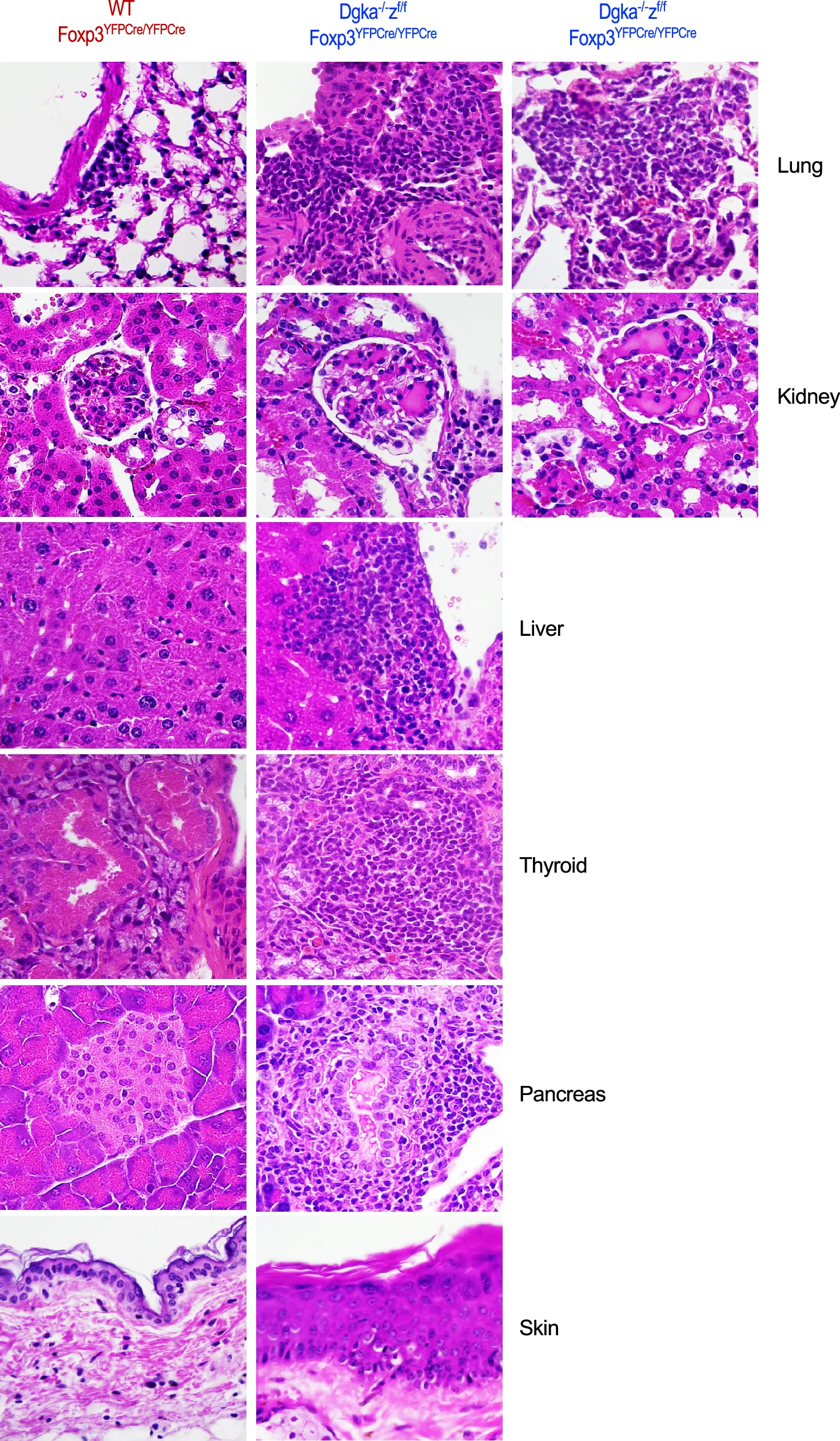
H&E staining of indicated organs correlating to Figure 1D with high magnification of the rectangle areas.

**Figure 1—figure supplement 2.**
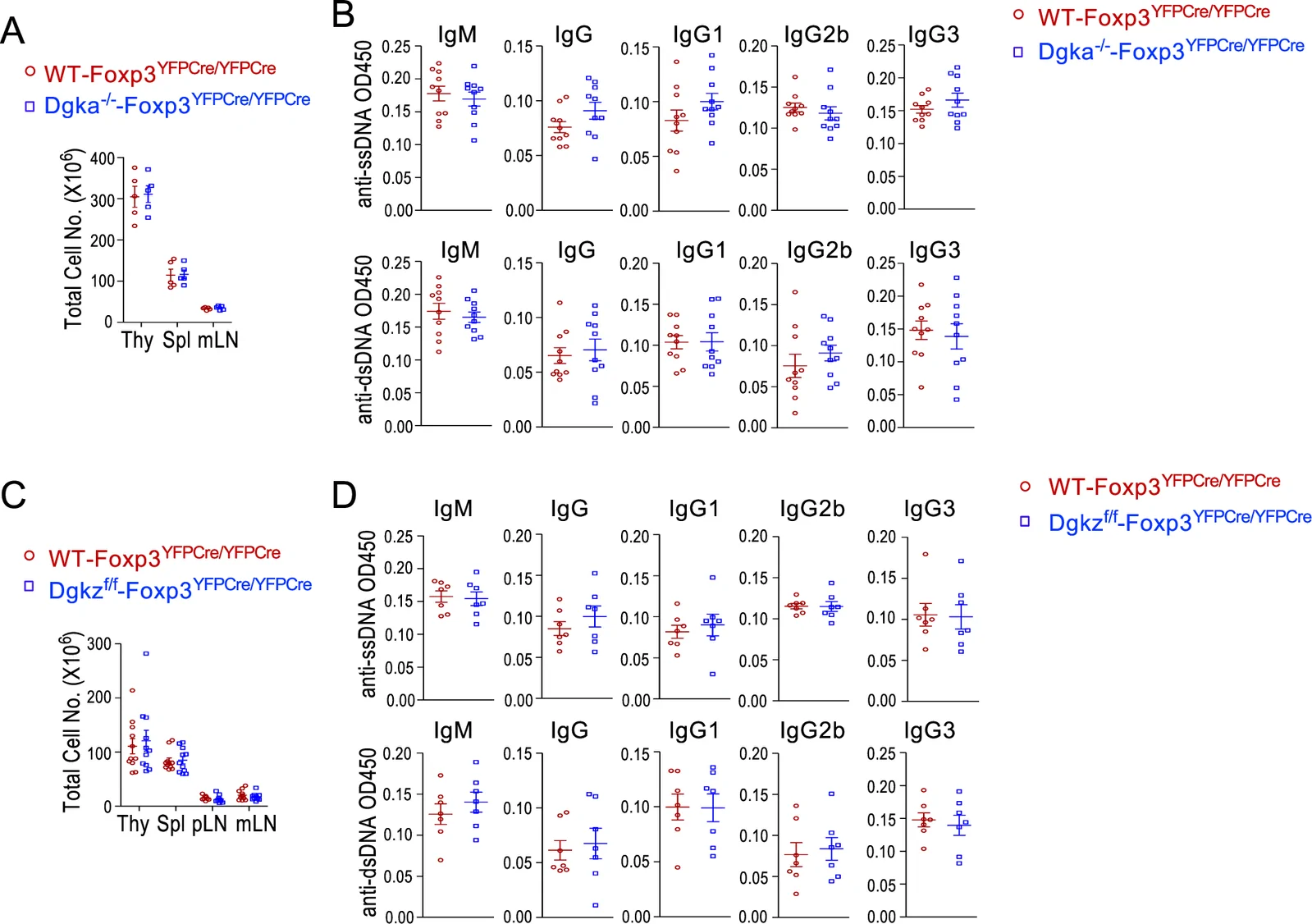
Analyses of *Dgka^-/-^-Foxp3^YFPCre/YFPCre^* mice and *Dgka^+/+^zf^f/f^-Foxp3^YFPCre/YFPCre^* mice. (**A–B**) *Dgka^-/-^-Foxp3^YFPCre/YFPCre^* mice and *WT-Foxp3^YFPCre/YFPCre^* mice. (**A**) Total cellularity. (**B**) Serum autoantibodies. (**C–D**) *Dgka^+/+^zf^f/f^-Foxp3^YFPCre/YFPCre^* mice and *WT-Foxp3^YFPCre/YFPCre^* mice. (**C**) Total cellularity. (**D**) Serum autoantibodies. Each circle or square represents one mouse of the indicated genotypes. Data shown are representative of or pooled from at least five experiments.

### Treg-specific DGKαζDKO enhances Treg homeostatic expansion

**Figure 2.**
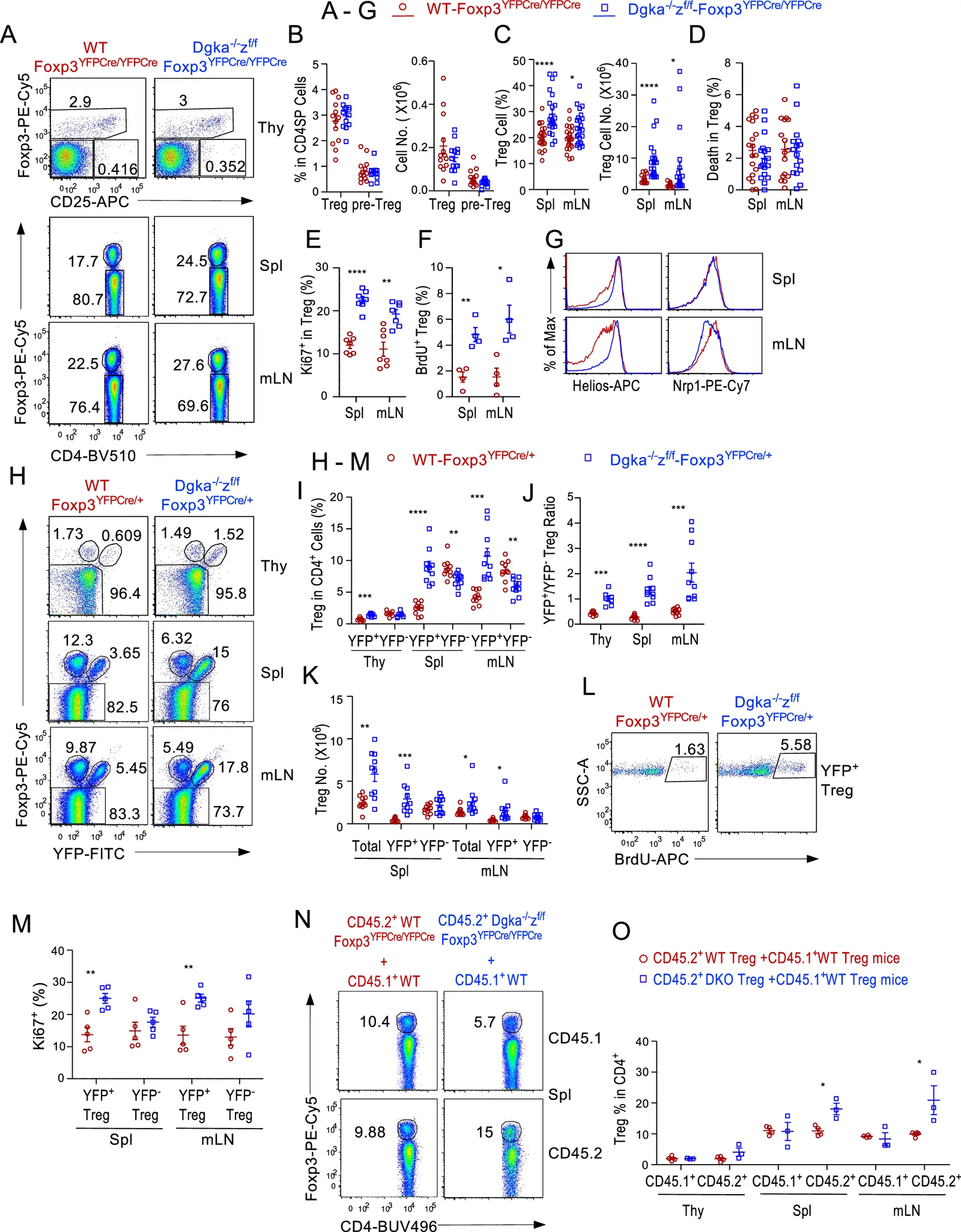
Enhanced Treg expansion in Treg-αζDKO mice. (**A–G**) Analysis of *Dgka^-/-^z^f/f^-Foxp3^YFPCre/YFPCre^* and WT*-Foxp3^YFPCre/YFPCre^* control mice. (**A**) Representative FACS plots showing Foxp3 and CD25 staining in CD4^+^ SP thymocytes (top panels) and CD4 and Foxp3 staining in splenic and LN CD4^+^ T cells. (**B**) Foxp3^+^ Treg and CD25^+^Foxp3^-^CD4^+^ pre-Treg percentages and numbers in the thymus. (**C**) Foxp3^+^ Treg percentages and numbers in the spleen and mLNs. (**D**) Death rate of Tregs. (**E**) Percentages of Ki67^+^ cells within Tregs. (**F**) BrdU incorporation in Tregs 8–10 hr after intraperitoneal (*i.p*.) injection of BrdU. (**G**) Overlaid histograms showing Helios and Nrp1 expression in Tregs. (**H–M**) Analysis of female *Dgka^-/-^z^f/f^-Foxp3^YFPCre/+^* and WT-*Foxp3^YFPCre/+^* mice. (**H**) Representative FACS plots showing intracellular Foxp3 and YFP staining in CD4^+^ T cells. (**I**) YFP^+^Foxp3^+^ and YFP^-^Foxp3^+^ Treg percentages in CD4^+^ T cells. (**J**) YFP^+^Foxp3^+^/YFP^-^Foxp3^+^ ratios in individual mice. (**K**) Total, YFP^+^, and YFP^-^ Treg numbers in the spleen and mLNs. (**L**) Representative FACS plots showing BrdU incorporation in LN YFP^+^ Tregs. (**M**) Percentages of Ki67^+^ cells in YFP^+^ and YFP^-^ Tregs. (**N, O**) Analyses of mixed BM chimeric mice. CD45.1^+^CD45.2^+^ WT mice were lethally irradiated and intravenously (*i.v*.) injected with a mixture of CD45.1^+^ WT with either CD45.2^+^ WT-*Foxp3^YFPCre/YFPCre^* or *Dgka^-/-^z^f/f^-Foxp3^YFPCre/YFPCre^* BM cells. Recipient mice were analyzed 6–8 weeks after reconstitution. (**N**) Intracellular staining of Foxp3 in CD4^+^TCRβ^+^ T cells. (**O**) CD45.1^+^CD45.2^-^ and CD45.1^-^CD45.2^+^ Treg percentages in individual mice. Data shown are representative of or pooled from 5 to 23 experiments except **F, L, N, and O**. (**F**) is pooled from four experiments. **L** represents two experiments. **N and O** are representative or pooled from three experiments. *, p<0.05; **, p<0.01; ***, p<0.001; ****, p<0.0001 determined by two-tailed unpaired Student's *t* test.

In Treg-αζDKO mice, CD4^+^Foxp3^+^ Tregs and CD4^+^CD25^+^Foxp3^-^ pre-Tregs were not altered in the thymus (Figure 2A B) but increased in the spleen and LNs (Figure 2). αζDKO Tregs survived similarly (Figure 2D) but increased in proliferation as indicated by Ki67 expression (Figure 2E) and BrdU incorporation (Figure 2F). They expressed slightly increased TF Helios, suggesting they were tTregs, although Nrp1 expression was similar or slightly reduced (Figure 2G). In contrast, Treg percentages and numbers were not altered in *Dgka^-/-^-Foxp3^YFPCre/YFPCre^* and *Dgka^+/+^zf^f/f^-Foxp3^YFPCre/YFPCre^* mice (Figure 2—figure supplement 1A - D).

**Figure 2—figure supplement 1.**
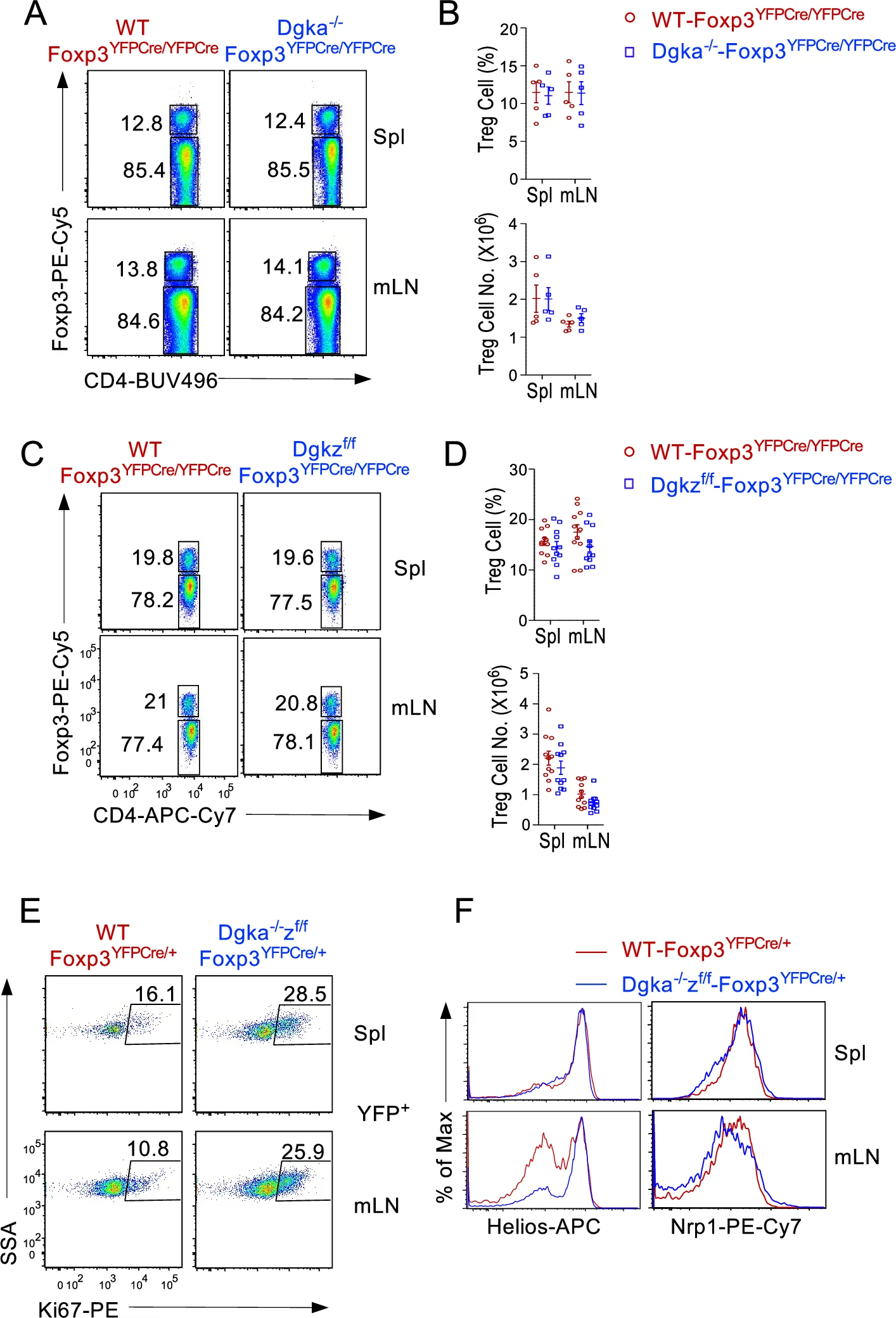
Analyses of Tregs in DGKa or DGKz single knockout mice. (**A, B**) Tregs in Dgka^-/-^ mice. (**C, D**) Tregs of *Dgka^+/+^zf^f/f^-Foxp3^YFPCre/YFPCre^* mice. (**E, F**) Analysis of female *Dgka^-/-^z^f/f^-Foxp3^YFPCre/+^* and *WT-Foxp3^YFPCre/+^* mice. (**E**) Ki67 staining in YFP^+^ Tregs. (**F**) Overlaid histograms showing Helios and Nrp1 expression in YFP^+^ Tregs. Each circle or square represents one mouse of the indicated genotypes. Data shown are representative of or pooled from at least five experiments.

In female *Dgka^-/-^z^f/f^-Foxp3^YFPCre/+^* (DKO-Cre^het^) mice that contained both YFP^-^Foxp3^+^*Dgka^-/-^z^wt^* Tregs to limit autoimmunity and YFP^+^Foxp3^+^αζDKO Tregs, YFP^+^Foxp3^+^ Treg percentages were increased in the thymus, spleen, and LNs, while YFP^-^Foxp3^+^ Tregs were decreased in the spleen and LNs (Figure 2H and I), leading to increased YFP^+^/YFP^-^ Treg ratios (Figure 2J). Moreover, female αζDKO-Cre^het^ mice had increased total and YFP^+^ but not YFP^-^ Treg numbers (Figure 2K) owing to hyperproliferation of YFP^+^ but not YFP^-^ Tregs (Figure 2L, M, Figure 2—figure supplement 1E). Additionally, YFP^+^αζDKO Tregs also expressed similar or increased levels of tTreg marker Helios (Figure 2—figure supplement 1F).

In lethally irradiated CD45.1^+^CD45.2^+^ recipient mice reconstituted with a mixture of CD45.1^+^ WT BM cells with either CD45.2^+^ WT or *Dgk*a*^-/-^z^f/f^-Foxp3^YFPCre/YFPCre^* BM cells, CD45.2^+^ Treg-αζDKO-derived Treg percentages within CD4^+^TCRβ^+^ cells were increased compared with CD45.1^+^ WT-derived Treg cells in the same recipient mice as well as with CD45.2^+^ WT-derived Tregs in CD45.2^+^ WT/CD45.1^+^ WT BM chimeric mice (Figure 2N and O).

Together, these data demonstrate that DGKα and ζ play an important, synergistic, and intrinsic role for Treg homeostasis by inhibiting Treg proliferation.

### Altered signaling, metabolic, and mTOR pathways and Th signatures in Tregs in Treg-αζDKO mice

**Figure 3.**
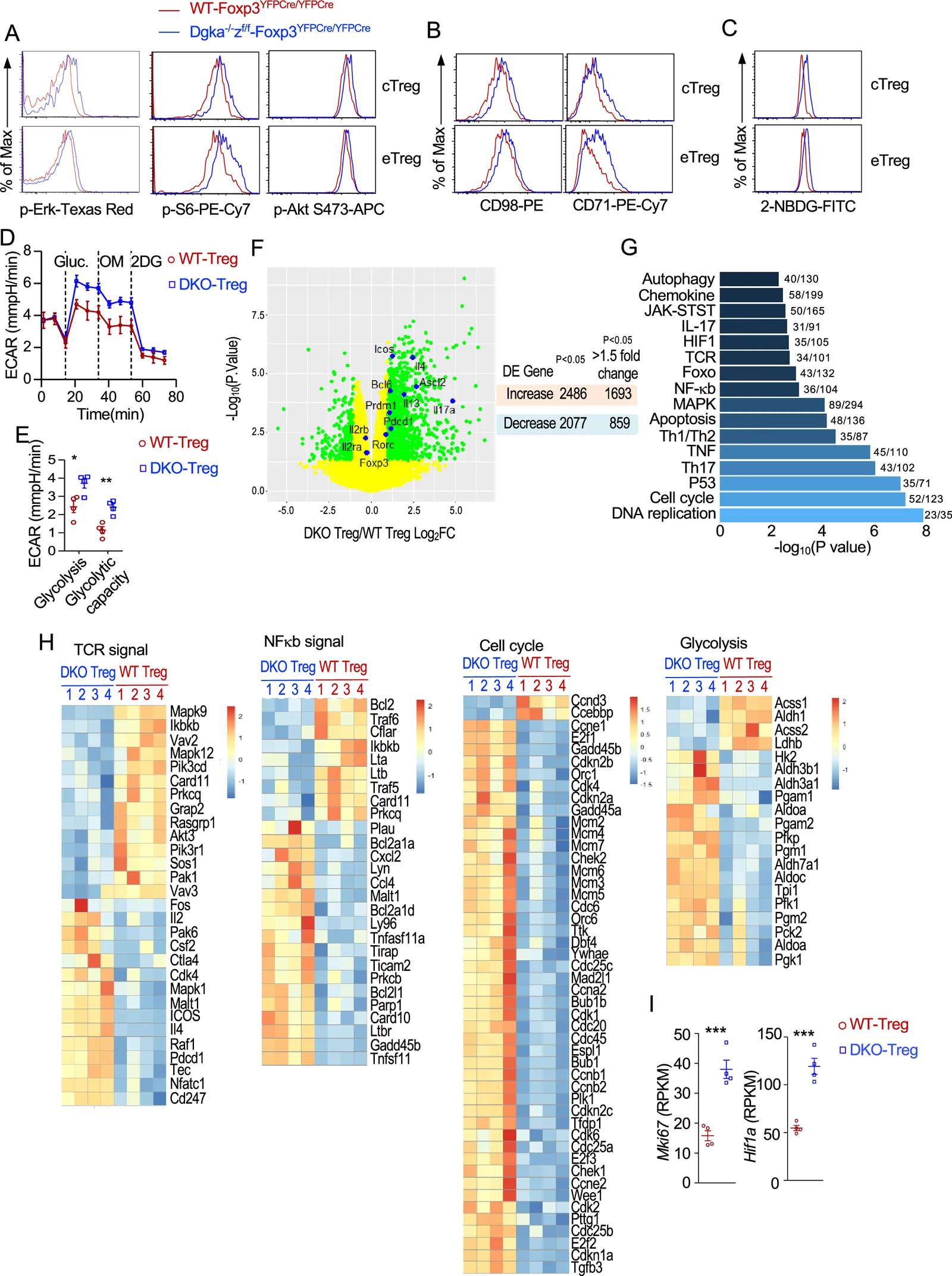
Altered signaling, metabolism, and transcriptional programs in αζDKO Tregs. Tregs in peripheral lymphoid organs from *Dgka^-/-^z^f/f^-Foxp3^YFPCre/YFPCre^* and WT*-Foxp3^YFPCre/YFPCre^* control mice were analyzed. (**A, B**) Overlaid histograms show Erk1/2, S6, and Akt S473 phosphorylation (**A**) and CD71 and CD98 expression (**B**) in cTregs and eTregs. (**C**) Overlaid histograms show 2-NBDG uptake in Tregs. (**D, E**) Extracellular acidification rate (ECAR) measurements of sorted Tregs (n=4 for both WT and DKO Tregs) following sequential treatment with glucose, oligomycin (OM, for mitochondrial perturbation), and 2DG (a glucose inhibitor). Representative ECAR profiles (**D**, WT, n = 4; DKO, n = 4) and summary scatter plot of ECAR (**E**). (**F**) Volcano plot comparison of gene expression between WT and DKO Tregs. Green-colored genes are differentially expressed with greater than 1.5-fold differences between WT and αζDKO Tregs (p<0.05). Right panel shows total numbers of DEGs in DKO Tregs. (**G**) Prominently changed KEGG pathways. (**H**) Heatmaps show DEGs in TCR signaling, NFκB, cell cycle, and glycolysis. (**I**) *Hif1a* and *Mki67* mRNA levels. *, p<0.05; **, p<0.01; ***, p<0.001 determined by two-tailed unpaired Student *t* test.

To understand how DGKαζDKO deregulated Tregs, we first examined signaling and metabolism in αζDKO-Tregs. αζDKO CD62L^+^CD44^low^ central (c) and CD62L^-^CD44^+^ effector (e) Tregs displayed increased Erk1/2, S6, and AktS473 phosphorylation (indications of enhanced Ras-Erk1/2, mTORC1, and mTORC2 signaling, Figure 3); elevated nutrient transporters CD71 and CD98 (Figure 3B); increased glucose uptake (Figure 3C); and enhanced glycolysis (Figure 3D and E). Thus, DGKα and ζ inhibited DAG-mediated Ras-Erk1/2 and mTOR signaling in Tregs and were important for proper Treg metabolism.

**Figure 3—figure supplement 1.**
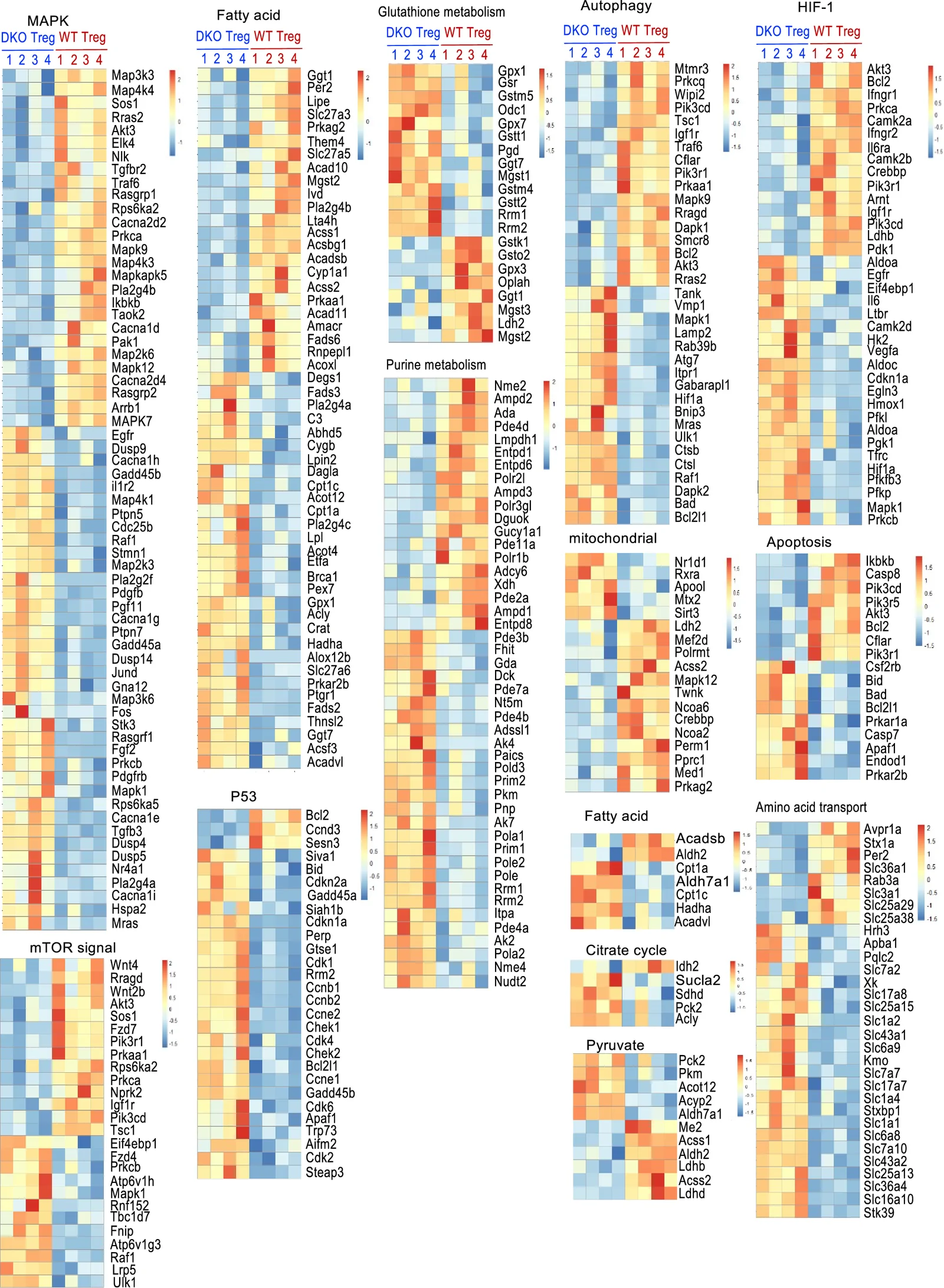
Heatmaps showing differentially expressed genes between WT and αζDKO Tregs in the indicated KEGG pathways.

We next performed transcriptomic analyses after sequencing RNA from WT*-* and *Dgka^-/-^z^f/f^-Foxp3^YFPCre/YFPCre^* CD4^+^Foxp3YFP^+^ Tregs. There were 4563 differentially expressed genes (DEGs, p<0.05) between these Tregs, with 2486 upregulated (1693>1.5-fold increase) and 2077 downregulated (859>1.5-fold decrease) in αζDKO-Tregs (Figure 3F, Supplementary file 1). KEGG pathway analyses revealed 132 enriched pathways (Figure 3G, Supplementary file 2), including those involved in cell expansion and homeostasis (DNA replication, cell cycle, P53, apoptosis, and autophagy pathways), Th differentiation and function (Th1/2/17, IL17, Foxo, cytokines, and cytokine signaling pathways, and Tfh pathway to be shown in Figure 5—figure supplement 1), TCR signaling and T cell activation (TCR, NFκB, MAPK, and cytokine pathways), metabolism (glycolysis, pyruvate, citrate cycle, glutathione, purine metabolism, fatty acid pathways, and nutrient transporters), and cell migration and homing (chemokines and their receptors, Figure 3H, Figure 3—figure supplement 1). αζDKO-Tregs upregulated many cell cycle-related genes including *Mki67* (Figure 3I), indicating that DGKαζ inhibited the cell cycle machinery to limit the normal Treg pool size. The enrichment of the glycolysis and Hif1 pathways, including upregulation of rate-limiting enzymes *Hk2* and *Pfk1* and *Hif1a* in DKO-Tregs (Figure 3G–I), suggested that DGKαζ inhibited glycolysis via multiple mechanisms.

In αζDKO-Tregs, several critical TCR signaling components such as *Vav2/3*, *Rasgrp1*, *Prkcq*, *Ikbkb, Card11*, *Pik3cd*, *Pik3r1*, *Akt3, and Pak1* were decreased, likely as a result of negative feedback mechanisms. Several other signal components, such as *Cd247* (encoding CD3ζ), *Tec*, *Raf1*, *Mapk1*, and *Malt1*, were increased. The enrichment of both MAPK and NFκB pathways (Figure 3H, Figure 3—figure supplement 1), coupled with enhanced Erk1/2 phosphorylation and mTORC1/2 activation (Figure 3A), indicated that DGKα and ζ prevented dysregulated DAG-mediated signaling in Tregs.

### Dysregulated eTreg differentiation, enhanced response to TCR stimulation, and altered properties of Treg-αζDKO Tregs

**Figure 4.**
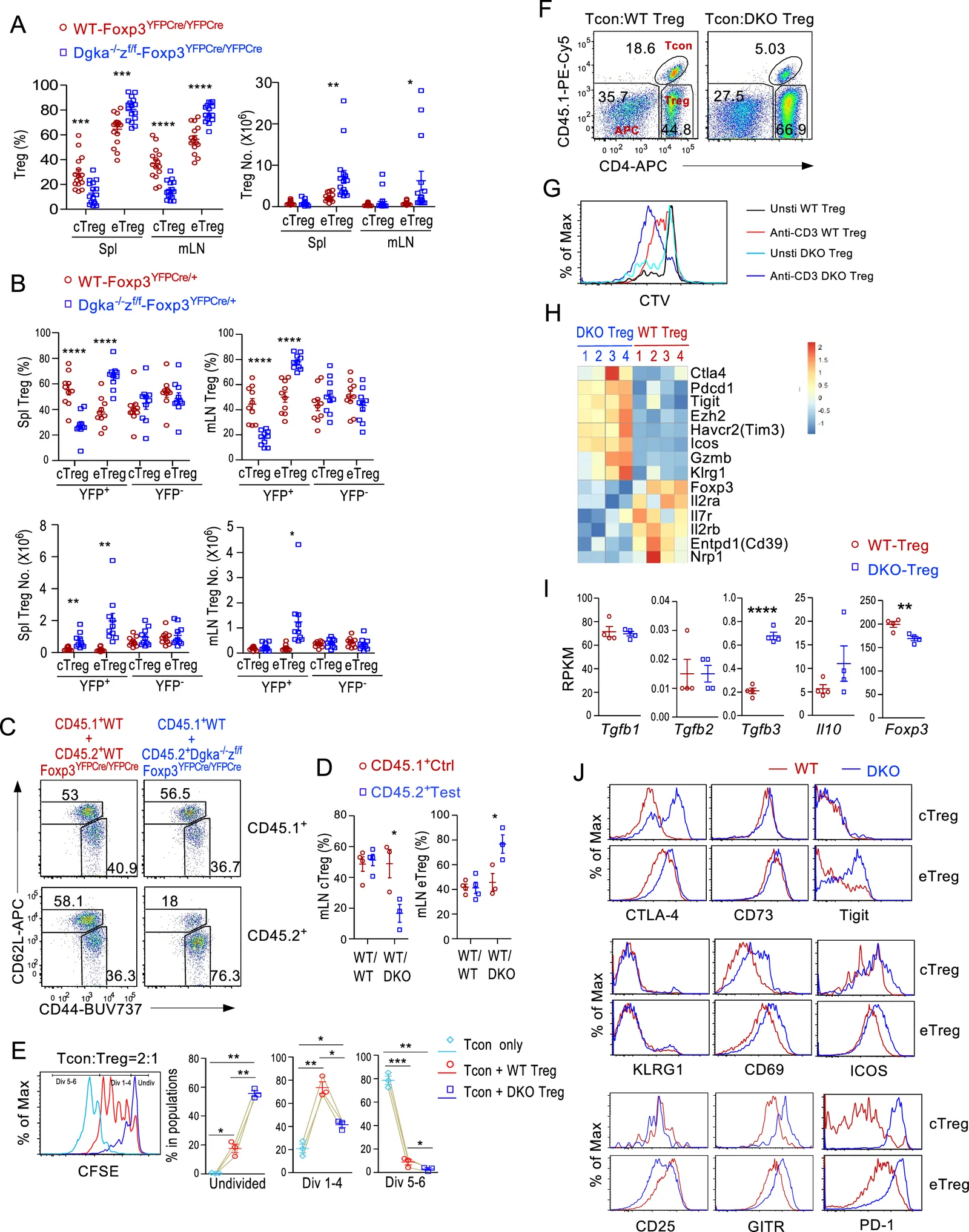
Enhanced effector differentiation and altered properties of αζDKO Tregs. (**A**) Scatter plots show mean ± SEM of cTreg and eTreg percentages and numbers in *Dgka^-/-^z^f/f^-Foxp3^YFPCre/YFPCre^* and WT*-Foxp3^YFPCre/YFPCre^* mice. (**B**) Scatter plots show mean ± SEM of cTreg and eTreg percentages and numbers of YFP^+^ and YFP^-^ CD4^+^Foxp3^+^ Tregs in female *Dgka^-/-^z^f/f^-Foxp3^YFPCre/+^* and WT*-Foxp3^YFPCre/+^* mice. (**C, D**) Analyses of mixed BM chimeric mice as described in Figure 2N. (**C**) CD44 and CD62L expression in CD45.1^+^ WT and in CD45.2^+^ WT or αζDKO Tregs in mLNs. (**D**) Scatter plots show mean ± SEM of cTreg and eTreg percentages in CD45.2^+^ and CD45.1^+^ Tregs. (**E, F**) In vitro contact inhibition assay. CTV labeled WT CD45.1^+^CD4^+^Foxp3YFP^-^ Tcon were mixed with a 2:1 ratio of CD45.1^-^CD45.2^+^ WT or αζDKO Tregs in the presence of mitomycin C treated splenocytes as antigen presenting cells (APCs) from TCRα^-/-^ mice and were stimulated with an anti-CD3 antibody for 72 hr. (**E**) Overlaid histograms show CTV dilution of CD45.1^+^ Tcon. Scatter plots show mean ± SEM of Tcon cells that were undivided, divided 1–4 times, and divided >5 times. (**F**) Tcon, Treg, and APC populations revealed by CD45.1 and CD4 expression. (**G**) TCR-induced Treg proliferation in vitro. Overlaid histogram showing CTV dilution of Foxp3YFP^+^ Tregs from CTV-labeled splenocytes after anti-CD3 stimulation for 72 hr. (**H**) Heatmap showing differentially expressed Treg effector molecules between WT and αζDKO Tregs (p<0.05). (**I**) mRNA levels of *Il10*, *Tgfb1-3*, and *Foxp3* in *Dgka^-/-^z^f/f^-Foxp3^YFPCre/YFPCre^* and WT-*Foxp3^YFPCre/YFPCre^* Tregs. (**J**) Overlaid histograms comparing expression of indicated molecules in *Dgka^-/-^z^f/f^-Foxp3^YFPCre/YFPCre^* and WT-*Foxp3^YFPCre/YFPCre^* mice. Data shown are representative of or pooled from at least three experiments. *, p<0.05; **, p<0.01; ***, p<0.001; ****, p<0.0001 determined by two-tail unpaired Student *t* test and pairwise Student *t* test for E.

Differentiation of cTregs to eTregs is important for immune suppression. In *Dgka^-/-^z^f/f^-Foxp3^YFPCre/YFPCre^* mice, cTreg and eTreg percentages were decreased and increased, respectively (Figure 4A, Figure 4—figure supplement 1A). Similar changes were also observed in YFP^+^ but not YFP^-^ Tregs in female *Dgka^-/-^z^f/f^-Foxp3^YFPCre/+^* mice (Figure 4B, Figure 4—figure supplement 1B) and in CD45.2^+^ *Dgka^-/-^z^f/f^-Foxp3^YFPCre/YFPCre^* Tregs in mixed BM chimeric mice (Figure 4C and D). The increased eTreg but decreased cTreg percentages were not due to decreases of cTreg numbers as cTreg numbers were not decreased in *Dgka^-/-^z^f/f^-Foxp3^YFPCre/YFPCre^* mice or even increased within YFP^+^ splenic Tregs in female *Dgka^-/-^z^f/f^-Foxp3^YFPCre/+^* mice (Figure 4A and B). Moreover, both cTregs and eTregs from *Dgka^-/-^z^f/f^-Foxp3^YFPCre/YFPCre^* mice showed enhanced proliferation (Figure 4—figure supplement 1C,D). The disproportional increases of αζDKO eTregs might be caused by accelerated cTreg to eTreg differentiation in the absence of both DGKα and ζ. However, a contribution of increased proliferation of αζDKO eTreg proliferation could not be ruled out. In contrast to Treg-αζDKO mice, c/eTreg ratios were not obviously altered in *Dgka^-/-^*- or *Dgka^+/+^zf^f/f^-Foxp3^YFPCre/YFPCre^* mice (Figure 4—figure supplement 2A-D). Thus, DGKα and ζ intrinsically and synergistically inhibited cTreg to eTreg differentiation.

**Figure 4—figure supplement 1.**
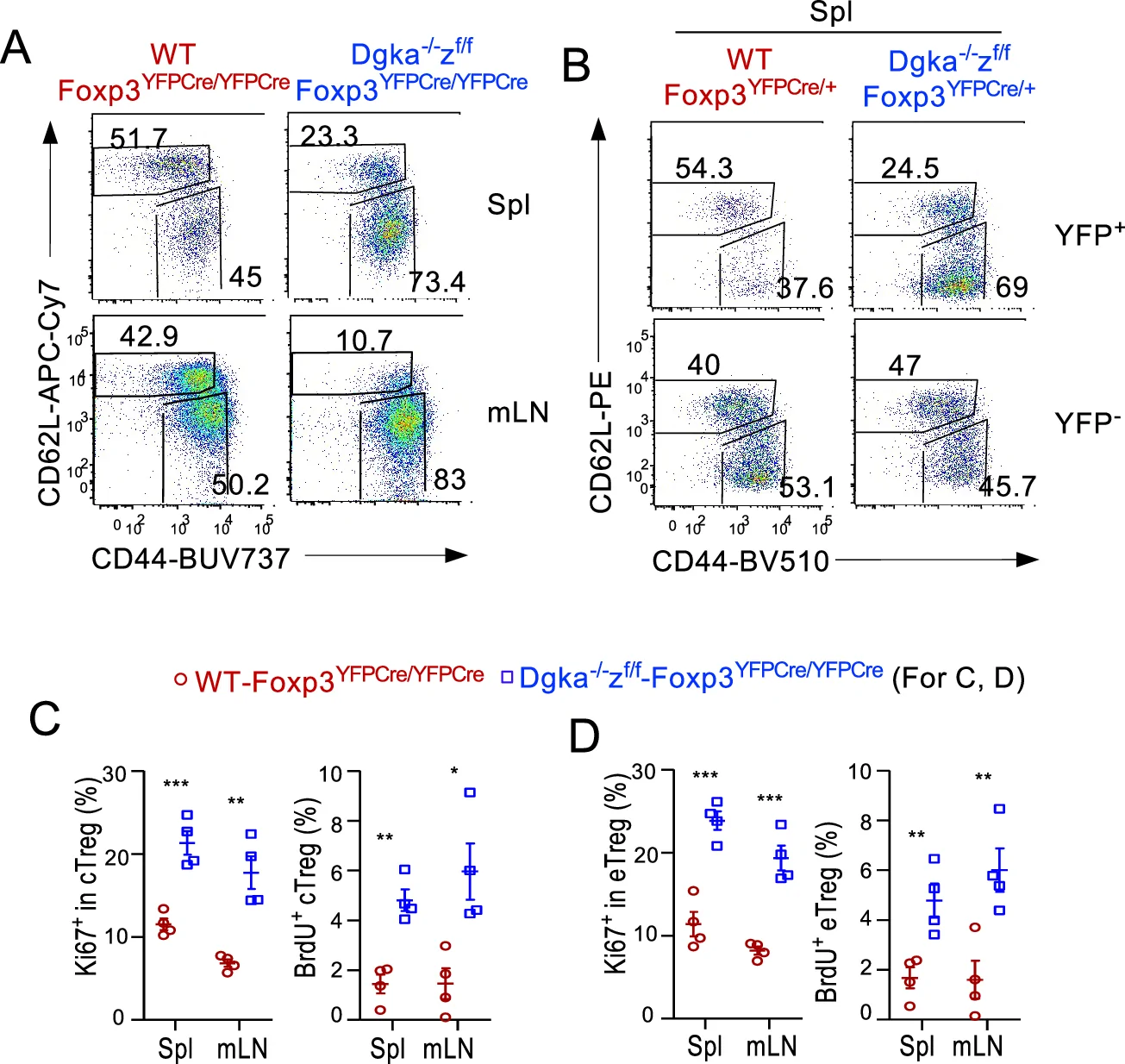
cTreg and eTreg ratios and proliferation in the absence of DGKalpha and zeta. (**A**) CD44 and CD62L expression in CD4^+^Foxp3^+^ Tregs from *Dgka^-/-^z^f/f^-Foxp3^YFPCre/YFPCre^* and *WT-Foxp3^YFPCre/YFPCre^* control mice. (**B**) CD44 and CD62L expression in YFP^+^ and YFP^-^ CD4^+^Foxp3^+^ Tregs from female *Dgka^-/-^z^f/f^-Foxp3^YFPCre/+^* and *WT-Foxp3^YFPCre/+^* control mice. (**C, D**) Scatter plots show mean ± SEM of percentages of Ki67+or BrdU + cells within cTregs (**C**) and eTregs (**D**).

**Figure 4—figure supplement 2.**
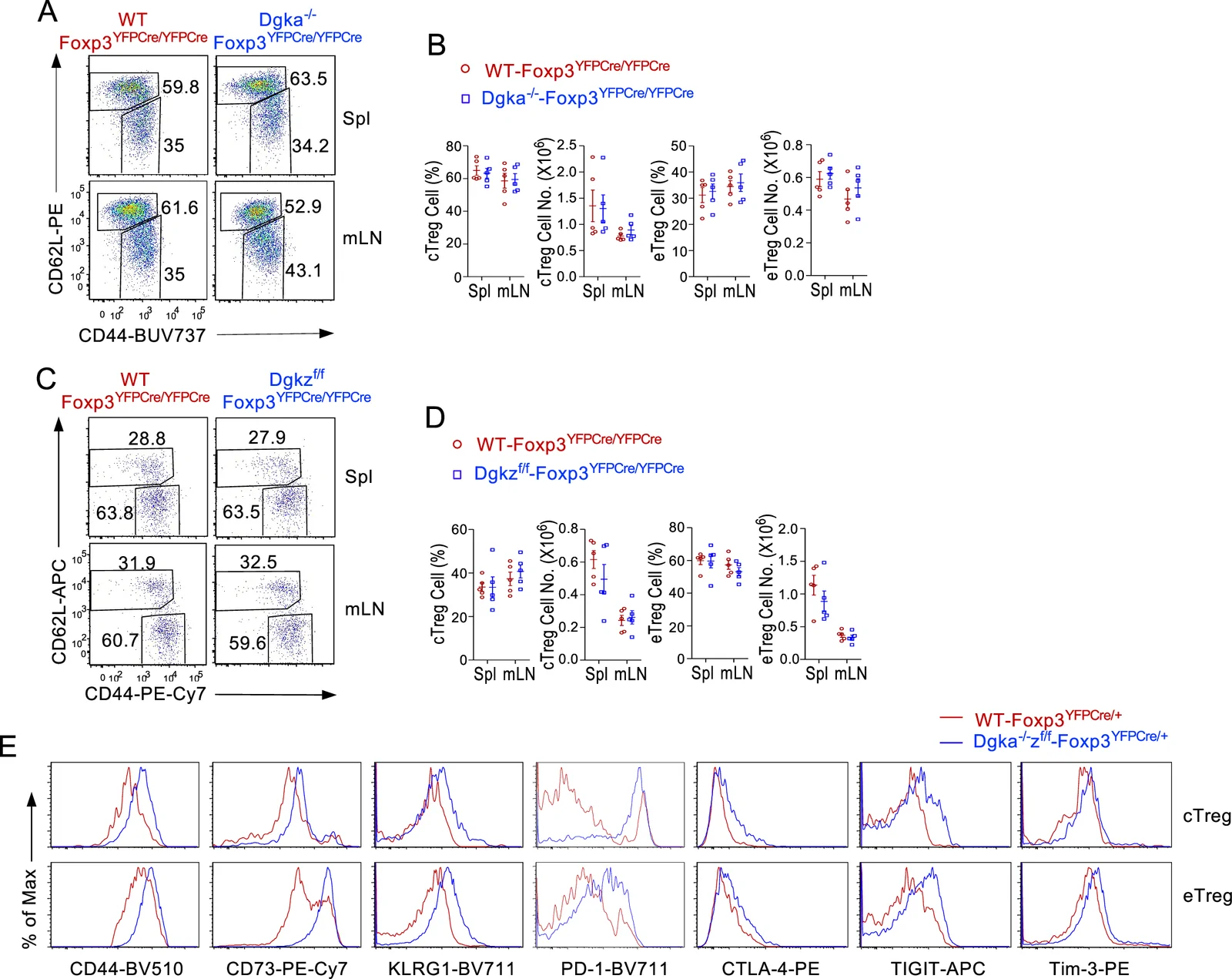
Treg properties in the absence of DGKa, z, or both. (**A, B**) Analyses of *Dgka^-/-^z^f/f^-Foxp3^YFPCre/YFPCre^* and *WT-Foxp3^YFPCre/YFPCre^* control mice. (**A**) CD44 and CD62L expression in CD4^+^Foxp3^+^ Tregs. (**B**) Scatter plots show mean ± SEM of cTreg and eTreg percentages and numbers. (**C, D**) Analyses of *Dgka^+/+^zf^f/f^-Foxp3^YFPCre/YFPCre^* and *WT-Foxp3^YFPCre/YFPCre^* control mice. (**C**) CD44 and CD62L expression in CD4^+^Foxp3^+^ Tregs. (**D**) Scatter plots show mean ± SEM of cTreg and eTreg percentages and numbers. (**E**) Overlaid histograms comparing expression of indicated molecules in YFP^+^ Tregs from female *Dgka^-/-^z^f/f^-Foxp3^YFPCre/+^* and *WT-Foxp3^YFPCre/+^* control mice. Data shown are representative of or pooled from at least five experiments.

αζDKO-Tregs displayed enhanced in vitro contact inhibition capability (Figure 4E), which is associated with their increased cell numbers after 72 hr incubation (Figure 4F). Moreover, αζDKO-Tregs proliferated more vigorously than WT-Tregs after in vitro TCR stimulation (Figure 4G). Thus, αζDKO-Tregs showed strong contact inhibition at least partially as a result of enhanced expansion. At present, it is unclear if individual αζDKO Treg exhibits stronger inhibition activity than a WT Treg.

αζDKO-Tregs manifested abnormal expression of many Treg-associated genes (Figure 4H and I). *Ctla4*, *Pdcd1* (encoding PD-1), *Tigit*, *Ezh2*, *Havcr2* (encoding Tim3), *Icos*, *Gzmb*, and *Klrg1* mRNA levels were increased, while *Foxp3*, *Il2ra*, *Il2rb*, *Il7ra*, *Entpd1* (encoding CD39), and *Nrp1* mRNA levels were decreased. At the protein level, CTLA-4, CD73, TIGIT, CD69, ICOS, GITR, and PD-1 were increased, and KLRG1 was not changed, but CD25 was decreased in either αζDKO cTregs, eTregs, or both (Figure 4J). Increased CTLA-4, TIGIT, GzmB, ICOS, and CD73, as well as *Tgfb3* and *Il10,* suggested that certain aspects of Treg function might be enhanced, while decreased C*d39*, *Il2ra*, *Il2rb*, *Il7r*, and *Ezh2* might negatively affect Treg function, homeostasis, and/or stability (Bayer et al., 2008; DuPage et al., 2015). The decreased *Foxp3* mRNA, although only 10%, and *Nrp1* mRNA levels might also contribute to or reflect reduced stability. Increased CD69 expression also supported enhanced DAG signaling. The drastically upregulated PD-1 in both cTregs and eTregs may have inhibited Treg function and raised the possibility of exhaustion. Some of these changes were also observed in *Dgka^-/-^z^f/f^-Fopx3^YFPCre/+^* female mice (Figure 4—figure supplement 2E), suggesting that DGKαζ double deficiency intrinsically influenced Treg properties.

### Altered Treg effector lineages and proinflammatory functions of αζDKO Tregs

**Figure 5.**
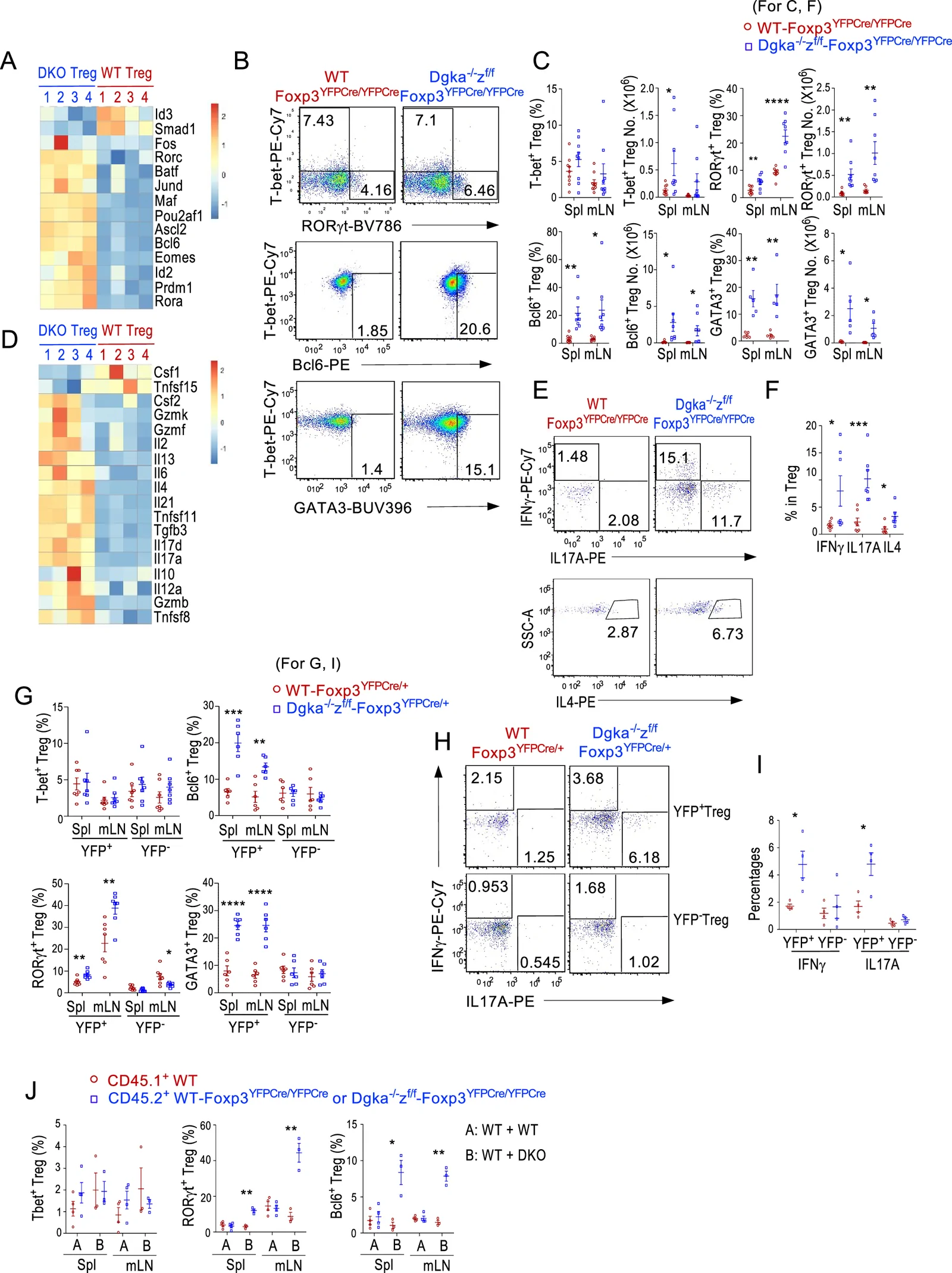
Increased effector lineages and gain-of-proinflammatory properties of αζDKO Tregs. (**A–F**) Analyses of WT-*Foxp3^YFPCre/YFPCre^* and *Dgka^-/-^z^f/f^-Foxp3^YFPCre/YFPCre^* Tregs. (**A**) Heatmap showing DE of TFs in Tregs by RNA sequencing. (**B**) Intracellular staining of RORγt, T-bet, Bcl6, and GATA3 in splenic Tregs. (**C**) Percentages and numbers (mean ± SEM) of Treg subsets. (**D**) Heatmap showing DE of cytokines in Tregs by RNA sequencing. (**E**) Intracellular staining of cytokines in Tregs after ex vivo PMA plus ionomycin stimulation in the presence of GolgiPlug for 5 hr. (**F**) Percentages (mean ± SEM) of IFNγ^+^, IL17A^+^, and IL4^+^ Tregs. (**G–I**) Analyses of female WT*-Foxp3^YFPCre/+^* and *Dgka^-/-^z^f/f^-Foxp3^YFPCre/+^* Tregs. (**G**) Percentages (mean ± SEM) of Treg effector sublineages in YFP^+^ and YFP^-^ Tregs. (**H–I**) IL17A and IFNγ expression in Tregs after ex vivo PMA plus ionomycin stimulation in the presence of GolgiPlug for 5 hr. (**H**) Representative FACS plots of Tregs. (**I**) Scatter plots show mean ± SEM of IFNγ^+^ and IL17A^+^ in YFP^+^ and YFP^-^ Tregs. (**J**) Analyses of mixed BM chimeric mice described in Figure 2N. Scatter plots show mean ± SEM of Treg sublineage percentages in CD45.1^+^ WT (red circle) and CD45.2^+^ WT or αζDKO Tregs (blue square). Data shown in B–J are representative of or pooled from at least three experiments. *, p<0.05; **, p<0.01; ***, p<0.001; ****, p<0.0001 determined by two-tail unpaired Student *t* test.

Tregs differentiate to multiple effector lineages to fulfill specific suppressive functions in different settings. T-bet^+^ Treg1, Gata3^+^, IRF4^+^, and Batf^+^ Treg2, RORγt^+^ and Stat3^+^ Treg17, and Bcl6^+^ Tfr-cells selectively suppress Th1, Th2, Th17, and Tfh cell/GC responses (Fu et al., 2018; Levine et al., 2017; Wang et al., 2011; Zheng et al., 2009; Chaudhry et al., 2009; Chung et al., 2011; Xu et al., 2021; Shan et al., 2023). As shown in Figure 5A, αζDKO-Tregs upregulated TFs associated with Th2 (*Maf* and *Batf*), Th17 (*Rora*, *Rorc*, *Maf*, *JunD*, *Pou2af1* [encoding Bob1/BOF1]), and Tfh (*Bcl6*, *Ascl2*, *Batf*, *Pou2af1*, and *Maf*) but not Th1 lineages (*Tbx21*). Increased RORγt and Bcl6 were further confirmed at the protein level by intracellular staining (Figure 5B, C, Figure 5—figure supplement 1A). Although *Gata3* mRNA was not increased, its protein was upregulated in αζDKO-Tregs, suggesting posttranscriptional regulation. Moreover, αζDKO-Tregs were enriched for Th2-, Th17-, and Tfh-like signatures (Figure 5—figure supplement 1B,C). Thus, differentiation of multiple Treg effector lineages, except Th1-like Tregs, was enhanced in Treg-αζDKO mice.

**Figure 5—figure supplement 1.**
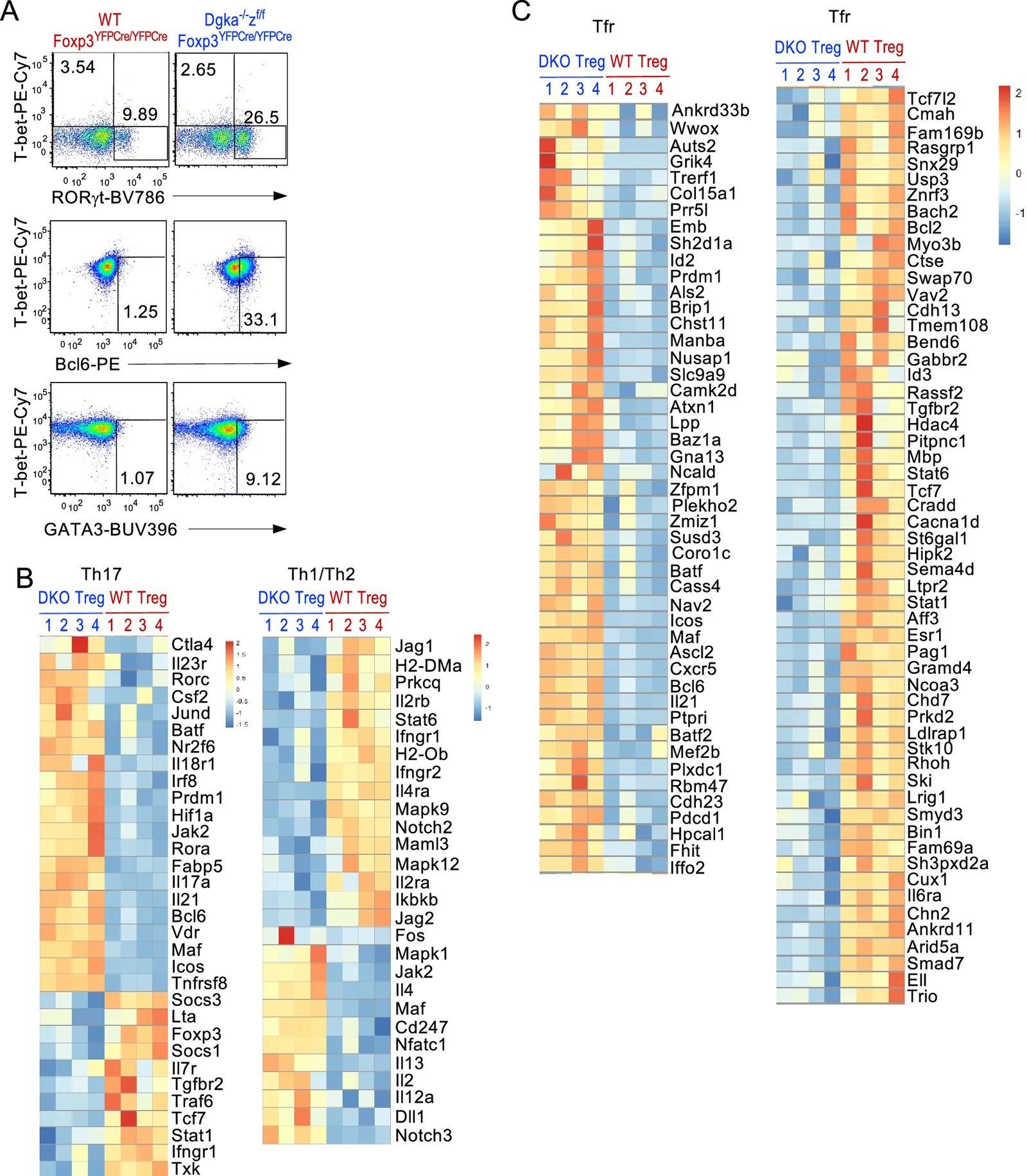
Analysis of effector lineages and gain-of-proinflammatory properties of αζDKO Tregs. (**A**) Intracellular staining of TF in mLN Tregs in *Dgka^-/-^z^f/f^-Foxp3^YFPCre/YFPCre^* and *WT-Foxp3^YFPCre/YFPCre^* mice. (**B, C**) Heatmaps showing altered Th1, Th2, and Th17 cell (**B**) and Tfr cell (**C**) associated genes with statistically significant differences between WT and αζDKO Tregs (p<0.05).

Because Tregs express TCRs with relatively high affinity to self-antigens, suppression of proinflammatory cytokine expression in Tregs is important for preventing Tregs from potential self-damage (Sakaguchi et al., 2008). Strikingly, αζDKO-Tregs expressed increased levels of *Il2*, Th2 cytokines *Il4* and *Il13*, Th17 cytokines *Il17a* and *Il17d*, Tfh cytokine *Il21*, proinflammatory cytokines *Il6* and *Csf2* (GM-CSF), and cytotoxicity-associated effectors *Gzmb*, *Gzmf*, and *Gzmk* (Figure 5D). Increased expression of IL17A and IL4 was further confirmed by intracellular staining (Figure 5E and F). Although T-bet and *Ifng* mRNA were not increased, IFNγ protein was increased, suggesting posttranscriptional regulation of IFNγ by DGKαζ.

The increases of GATA3^+^, RORγt^+^, and Bcl6^+^ sublineages and elevated IL17A and IFNγ production in αζDKO-Tregs were also observed in the YFP^+^ Tregs in female *Dgka^-/-^z^f/f^-Foxp3^YFPCre/+^* mice (Figure 5G–I) as well as in the CD45.2^+^ αζDKO-Treg in mixed BM chimeric mice (Figure 5J).

Together, these data revealed that DGKα and ζ intrinsically suppress Treg effector lineage differentiation and also inhibit expression of multiple cytokines including Th effector cytokines IL-4, IL-17, and IFNγ in Tregs at transcriptional and posttranscriptional levels.

### Deregulated effector functions of CD4^+^Foxp3^-^ and CD8^+^ T cells in Treg-αζDKO mice

**Figure 6.**
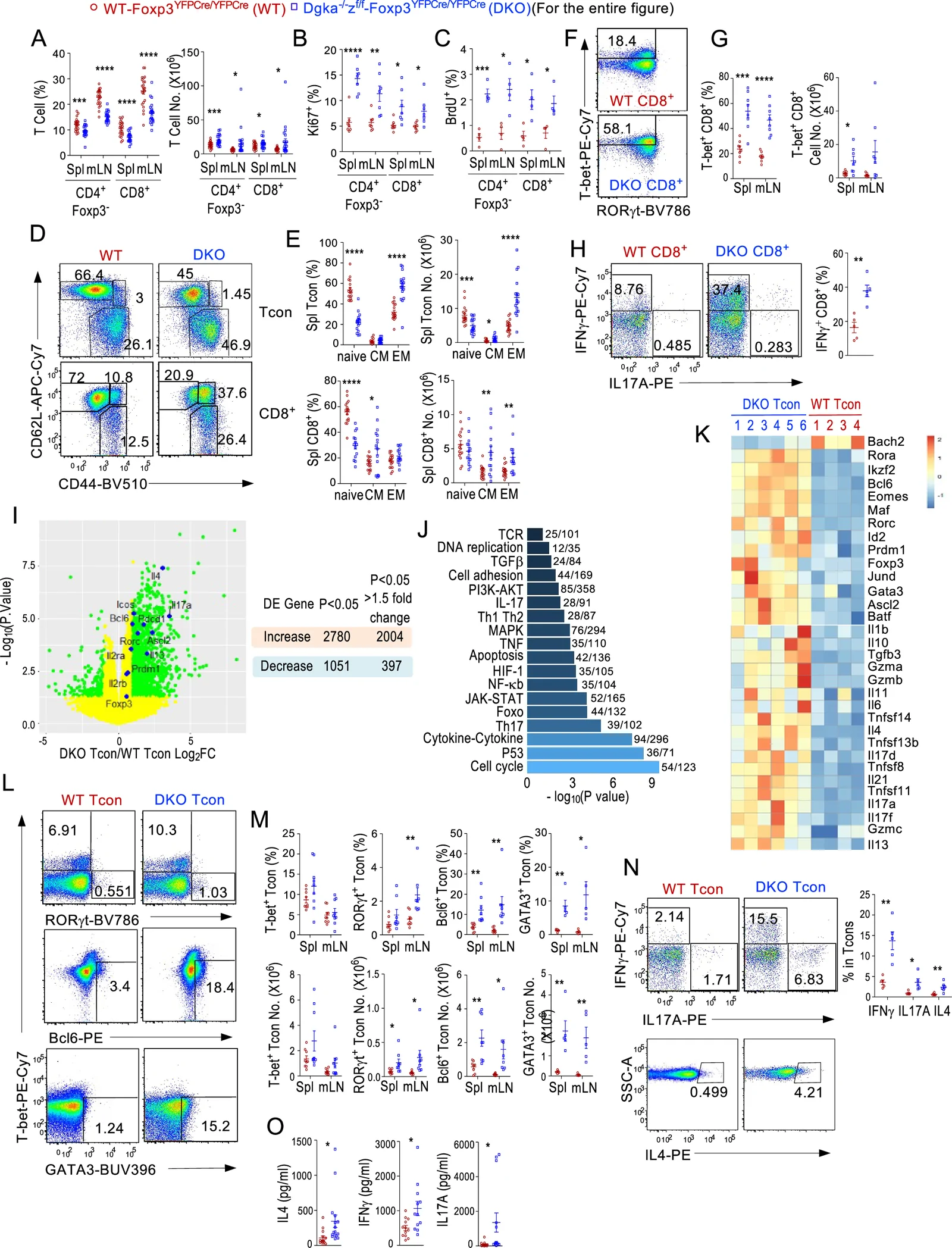
Enhanced effector function of CD4^+^Foxp3^-^ Tcon and CD8 T cells in Treg-αζDKO mice. *Dgka^-/-^z^f/f^-Foxp3^YFPCre/YFPCre^* and WT*-Foxp3^YFPCre/YFPCre^* control mice were analyzed. (**A**) CD4^+^Foxp3^-^ Tcon and CD8^+^ T cell percentages and numbers in the spleen and mLNs. (**B**) Ki67^+^ cells in Tcon and CD8 T cells. (**C**) BrdU^+^ cells in Tcon and CD8 T cells. (**D**) Representative FACS plots showing CD44 and CD62L expression in splenic CD4^+^Foxp3^-^ Tcon and CD8^+^ T cells. (**E**) Naïve, CM, and EM percentages and numbers of splenic CD4^+^Foxp3^-^ Tcon and CD8 T cells. (**F**) Representative FACS plots showing intracellular T-bet and RORγt staining in LN CD8 T cells. (**G**) Percentages and numbers of T-bet^+^ CD8 T cells. (**H**) Intracellular IFNγ and IL17A staining in CD8 T cells after PMA and ionomycin stimulation. Scatter plot represents mean ± SEM of IFNγ^+^ CD8 T cells from 6- to 12-month-old mice. (**I**) Volcano plot comparing mRNA expression in WT and Treg-αζDKO CD4^+^Foxp3^-^ Tcons after RNA-seq analysis. Genes colored in green are differentially expressed with greater than 1.5-fold differences between WT and αζDKO Tregs (p<0.05). Table shows numbers of DE genes (p<0.05). (**J**) Top enriched KEGG pathways between WT and Treg-αζDKO CD4^+^Foxp3^-^ Tcons. (**K**) Heatmap showing mRNA levels of TFs and cytokines that were differentially expressed between WT and Treg-αζDKO CD4^+^Foxp3^-^ Tcons (p<0.05). (**L**) T-bet, RORγt, Bcl6, and GATA3 proteins in splenic Tcons detected by intracellular staining. (**M**) Percentages and numbers of T-bet^+^, RORγt^+^, Bcl6^+^, and GATA3^+^ cells in Tcons. (**N**) IFNγ, IL17A, and IL4 protein levels in WT and Treg-αζDKO CD4^+^Foxp3^-^ Tcons detected by intracellular staining after PMA and ionomycin stimulation for 5 hr. (**O**) Serum cytokine levels in 5- to 12-month-old WT and Treg-αζDKO mice. Data shown are representative of or pooled from 4 to 22 experiments. *, p<0.05; **, p<0.01; ***, p<0.001; ****, p<0.0001 determined by two-tail unpaired Student *t* test.

**Figure 7.**
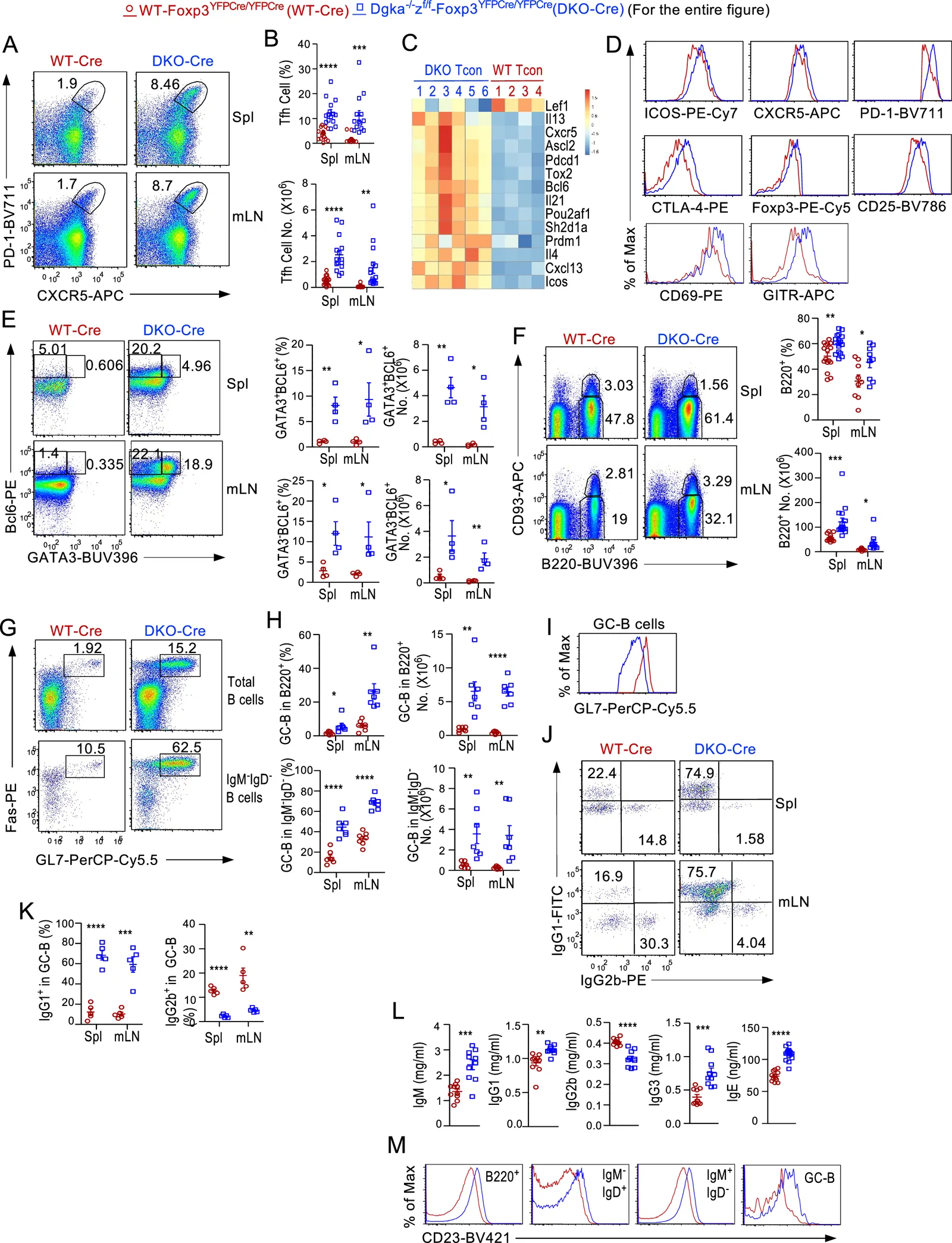
Enhanced Tfh/GC-B cell responses in *Dgka^-/-^z^f/f^-Foxp3^YFPCre/YFPCre^* mice. Splenocytes and LN cells from *Dgka^-/-^z^f/f^-Foxp3^YFPCre/YFPCre^* and WT-*Foxp3^YFPCre/YFPCre^* mice were analyzed. (**A**) CXCR5 and PD-1 expression in CD4^+^Foxp3^-^ Tcons. (**B**) Percentages and numbers of CXCR5^+^PD-1^+^ Tfh-cells in 2- to 14-month-old mice. (**C**) Heatmap shows DE of key Tfh/Tfr genes from transcriptomic analyses of Tcons described in Figure 6I. (**D**) Overlaid histograms show expression-indicated molecules in Tfh cells. (**E**) Representative FACS plots show GATA3 and Bcl6 expression in Tcons from a pair of 7-month-old mice. Scatter plots show mean ± SEM of GATA3^+^Bcl6^+^ and GATA3^-^Bcl6^+^ Tfh cells. (**F**) B220 and CD93 staining of splenocytes and LN cells. Scatter plots show mean ± SEM of B220^+^CD93^-^ mature B-cell percentages and numbers. (**G**) GL7 and Fas expression in total B220^+^ and in IgM^-^IgD^-^B220^+^ cells. (**H**) Scatter plots show mean ± SEM of GC-B cell percentages and numbers in 5- to 14-month-old mice. (**I**) Overlaid histogram shows GL7 expression in GC-B cells. (**J**) Intracellular IgG1 and IgG2b staining in GC-B cells. (**K**) IgG1^+^ and IgG2b^+^ percentages in GC-B cells. (**L**) Serum IgM, IgG1, IgG2b, IgG3, and IgE concentrations. (**M**) CD23 expression in B cell populations. Data shown are representative of or pooled from at least four experiments. *, p<0.05; **, p<0.01; ***, p<0.001; ****, p<0.0001 determined by two-tail unpaired Student *t* test.

Abnormal Treg functions could lead to deregulation of CD4^+^Foxp3^-^ conventional T cells (Tcons) and CD8^+^ T-cells. In Treg-αζDKO mice, CD4^+^Foxp3^-^ Tcons and CD8^+^ T-cells were increased in number in spleen and LNs (Figure 6A) at least partially as a result of enhanced proliferation (Figure 6B and C), although their percentages were decreased owing to disproportional expansion of B-cells, as detailed in Figure 7F. They contained decreased naïve but increased CD44^+^CD62L^+^ central memory (CM) and/or CD44^+^CD62L^-^ effector memory (EM) cells (Figure 6D and E), accompanying increased T-bet^+^ and IFNγ-producing CD8^+^ T-cells (Figure 6F–H), suggesting impaired Treg suppression on CD8^+^ T-cells in these mice.

**Figure 6—figure supplement 1.**
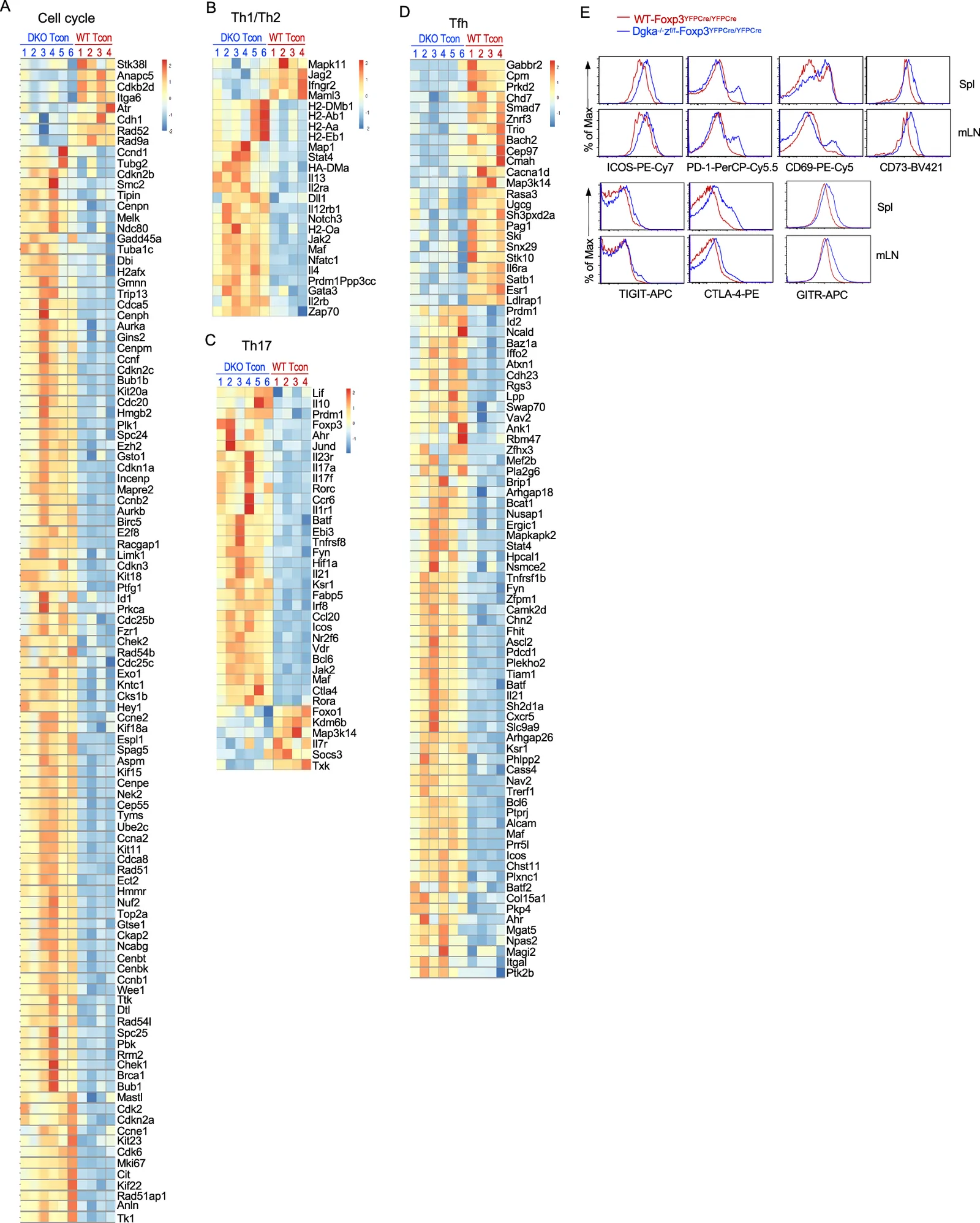
Altered cell cycle and Th associated pathways in CD4+Foxp3- Tcons from Treg-azDKO mice. (**A**) Heatmap shows DE cell cycle associated genes. (**B**) Heatmap shows DE Th1/2 associated genes. (**C**) Heatmap shows DE Th17 associated genes. (**D**) Heatmap shows DE Tfh/Tfr associated genes. (**E**) Altered expression of surface molecules in Tcons from *Dgka^-/-^z^f/f^-Foxp3^YFPCre/YFPCre^* and *WT-Foxp3^YFPCre/YFPCre^* mice.

**Figure 7—figure supplement 1.**
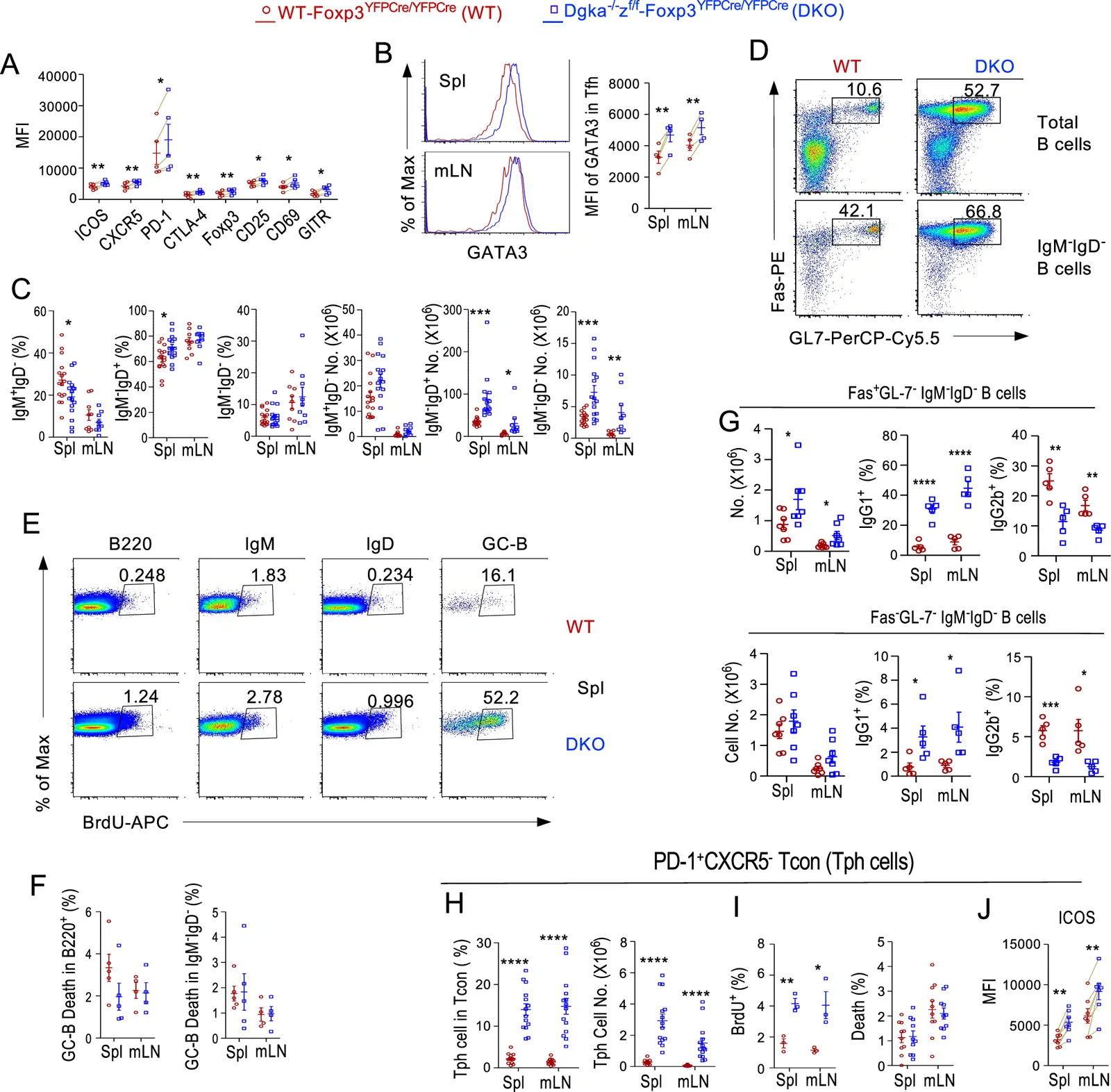
Assessment of Tfh, Tph and B cells in *Dgka^-/-^z^f/f^-Foxp3^YFPCre/YFPCre^* and *WT-Foxp3^YFPCre/YFPCre^* mice. (**A**) Scatter plot shows mean ± SEM of MFI of the indicated molecules in Tfh cells. (**B**) GATA3 levels in Tfh cells. (**C**) IgM^+^IgD^-^, IgM^-^IgD^+^, and IgM^-^IgD^-^ B cell percentages and numbers. (**D**) GC-B cell staining in mLNs. (**E**) BrdU incorporation in B cell populations. (**F**) Death rates of GC-B cells. (**G**) Fas^+^GL7^-^ and Fas^-^GL7^-^ IgM^-^IgD^-^ B cell numbers and IgG1^+^ or IgG2b^+^ ratios. (**H – J**) CD4^+^TCRβ^+^Foxp3^-^PD1^+^CXCR5^-^ Tph cell percentages and numbers (**H**), BrdU incorporation and death rate (**I**), and ICOS levels (**J**). Each circle or square represents one mouse of the indicated genotypes. Data shown are representative of or pooled from at least four experiments. *, p<0.05; **, p<0.01; ***, p<0.001; ****, p<0.0001 determined by two-tail unpaired Student t test (**C, F, G – I**) or pairwise Student t-test for **A, B, and J**.

**Figure 7—figure supplement 2.**
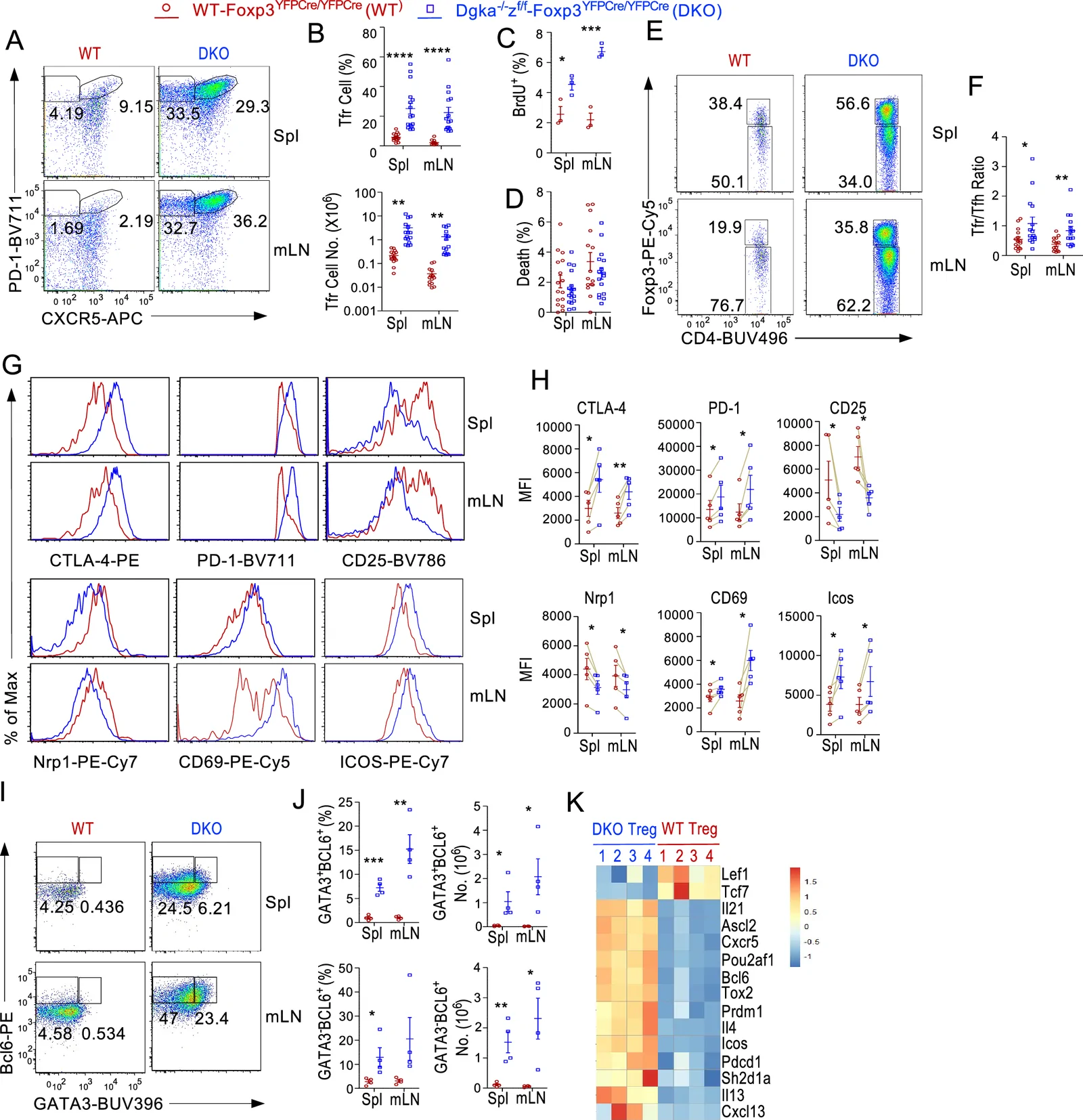
Dysregulation of Tfr cells in *Dgka^-/-^z^f/f^-Foxp3^YFPCre/YFPCre^* mice. Splenocytes and LN cells from *Dgka^-/-^z^f/f^-Foxp3^YFPCre/YFPCre^* and *WT-Foxp3^YFPCre/YFPCre^* mice were analyzed. (**A**) CXCR5 and PD-1 expression in CD4^+^Foxp3^+^ Tregs. (**B**) Mean ± SEM of Tfr percentages and numbers. (**C**) BrdU incorporation in Tfr cells. (**D**) Death rates of Tfr cells. (**E**) Foxp3 and CD4 expression in CXCR5^+^PD-1^+^ CD4^+^ T cells. (**F**) Mean ± SEM of Tfr/Tcon ratios. (**G**) Representative histograms show expression of indicated molecules in Tfr cells. (**H**) Scatter plots represent mean ± SEM of MFI of the indicated molecules in Tfr cells. (**I**) Bcl6 and GATA3 expression in CD4^+^Foxp3^+^ Tregs. (**J**) Mean ± SEM of percentages and numbers of GATA3^+^Bcl6^+^ and GATA3^-^Bcl6^+^ cells in Tregs. (**K**) Heatmap showing expression of Tfr-/Tfh-cell-related genes in WT and DKO Tregs. Data shown are representative of or pooled from 3 to 16 experiments. *, p<0.05; **, p<0.01; ***, p<0.001; ****, p<0.0001 determined by two-tail unpaired Student t test (**B – D**, **F and J**) or pairwise Student t-test for H.

To facilitate the understanding of abnormalities in CD4^+^ Tcons, we performed transcriptomic analyses of CD4^+^Foxp3YFP^-^ Tcons from WT*-Foxp3^YFPCre/YFPCre^* and *Dgka^-/-^z^f/f^-Foxp3^YFPCre/YFPCre^* mice. Because CD4^+^Foxp3YFP^-^ Tcons from *Dgka^-/-^z^f/f^-Foxp3^YFPCre/YFPCre^* mice should contain WT (more accurately, DGKα-deficient) Tcons and DGKαζ double-deficient ex-Foxp3 cells (exTregs), we reasoned that such analyses would be informative for revealing abnormalities of Tcons resulting from impairment of certain aspects of Treg functions and potential gained properties of exTregs arising from absence of both DGKα and ζ.

Treg-αζDKO CD4^+^Foxp3^-^ Tcon transcriptome displayed obvious differences from control Tcon's. They had 2780 upregulated and 1051 downregulated genes (p<0.05, Supplementary file 3) including 2004 and 397 of them respectively with differences greater than 1.5-fold (Figure 6I). One hundred and thirty-four KEGG pathways, with striking similarities to those of Tregs, were enriched in αζDKO Tcons (Figure 6J, Supplementary file 4). Cell cycle and DNA replication were also among the top enriched pathways with many cell cycle-promoting molecules upregulated (Figure 6—figure supplement 1A), further supporting dysregulated Tcon expansion.

Treg-αζDKO CD4^+^Foxp3^-^ Tcons were also enriched with Th1, Th2, Th17, and Tfh signatures (Figure 6J, K, Figure 6—figure supplement 1B-D). mRNA levels of multiple TFs associated with effector differentiation (*Eomes*, *Prdm1* [encoding Blimp1], and *Klrg1*), Th2 differentiation (*Gata3*, *cMaf, Batf*), Th17 differentiation (*Rora*, *Rorc*, *cMaf*, and *Jund*), and Tfh differentiation (*Bcl6*, *Batf*, *Ascl2*, and *Pou2af1*) were increased (Figure 6K). Expression of *Bach2*, which inhibits Tfh and Th17 differentiation to promote Treg stability (Roychoudhuri et al., 2013), was decreased. Consistently, RORγt^+^, Bcl6^+^, GATA3^+^, and, to a lesser extent, T-bet^+^ cells within Treg-αζDKO CD4^+^Foxp3^-^ Tcons were increased (Figure 6L and M), accompanying increased mRNA of many Th2 (*Il4* and *Il13*), Th17 (*Il17a/d/f*), and Tfh (*Il21*) cytokines (Figure 6K) and IL4, IL17A, and IFNγ proteins (Figure 6N). In some old Treg-DKO mice, serum IL4, IFNγ, and IL17A levels were elevated (Figure 6O). CD4^+^Foxp3^-^ T cells from *Dgka^-/-^z^f/f^-Foxp3^YFPCre/YFPCre^* expressed altered cell surface markers. They had upregulated CTLA-4, ICOS, PD-1, CD73, TIGIT, and several other molecules (Figure 6—figure supplement 1E). Thus, Treg-αζDKO CD4 Tcons, remarkably similar to Tregs, displayed enhanced Th2/17 and Tfh differentiation.

Together, these data indicated that deficiency of both DGKα and ζ in Tregs led to enhanced proliferation and effector functions of both CD4^+^Foxp3^-^ Tcons and CD8^+^ T-cells. Such abnormalities might play a role in the overall inflammatory and autoimmune status in Treg-αζDKO mice and contribute to the phenotypes we observed in these mice.

### Enhanced Tfh- and Tfh2/13-skewed GC responses in Treg-αζDKO mice

Tfh-cells are crucial for GC responses and humoral immunity. In *Dgka^-/-^z^f/f^-Foxp3^YFPCre/YFPCre^* mice, Foxp3^-^CD4^+^CXCR5^+^PD-1^+^ Tfh-cell percentages and numbers were drastically increased (Figure 7A and B), which was consistent with the enrichment of the Tfh pathway and increased expression of Tfh TFs *Bcl6*, *Ascl2*, and *Pou2af1* (Hatzi et al., 2015; Liu et al., 2014; Stauss et al., 2016) and effector molecules *Il21*, *Il4*, and *Il13* in CD4^+^Foxp3^-^ Tcons (Figures 6K-M, 7C,, Figure 6—figure supplement 1D). Treg-αζDKO Tfh cells upregulated Tfh-promoting molecule ICOS and CXCR5; T-cell activation markers CD69 and PD-1; and Treg-associated molecules CTLA-4, CD25, GITR, and Foxp3 (Figure 7D, Figure 7—figure supplement 1A), with both GATA3^+^Bcl6^+^ IL4/IL13-expressing Tfh2/13 cells (Gowthaman et al., 2019) and GATA3^-^Bcl6^+^ Tfh-cells increasing (Figure 7E) and elevated GATA3 levels in Tfh cells (Figure 7—figure supplement 1B).

Consistent with increased Tfh-cells, *Dgka^-/-^z^f/f^-Foxp3^YFPCre/YFPCre^* mice had increased B220^+^ B-cells, mostly as a result of increases of IgM^-^IgD^+^ and IgM^-^IgD^-^ cells (Figure 7F, Figure 7—figure supplement 1C) and increased GL7^+^Fas^+^ GC-B-cells and GL7^-^Fas^+^ activating B-cells within total B220^+^ or IgM^-^IgD^-^B220^+^ cells (Figure 7G, H, Figure 7—figure supplement 1D). GC-B cells from Treg-αζDKO mice showed decreased GL7 levels, likely reflecting overactivation (Figure 7I), enhanced proliferation but similar survival (Figure 7—figure supplement 1E,F), and markedly increased IgG1^+^ but reduced IgG2b^+^ ratios (Figure 7J and K). Additionally, IgM^-^IgD^-^FAS^+^GL-7^-^ and IgM^-^IgD^-^FAS^-^GL-7^-^ non-GC B cell numbers were increased and both populations contained increased IgG1 but decreased IgG2b ratios (Figure 7—figure supplement 1G). Consistently, CD4^+^Foxp3^-^ CXCR5^-^PD-1^+^ T peripheral helper (Tph) cells, which promote extrafollicular B cell antibody responses and contribute to autoimmunity (Rao et al., 2017; Yoshitomi and Ueno, 2021; Bocharnikov et al., 2019; Ekman et al., 2019), were increased in both percentages and numbers (Figure 7—figure supplement 1), associated with increased proliferation but not survival (Figure 7—figure supplement 1I) and upregulated ICOS levels (Figure 7—figure supplement 1J). Treg-αζDKO mice contained increased serum IgM, IgG1, IgE, and IgG3 levels but decreased IgG2b levels (Figure 7L), accompanying obviously increased CD23 (the low affinity receptor for IgE) in various B-cells (Figure 7M), which is consistent with the ability of serum IgE to upregulate CD23 (Selb et al., 2017).

Together, DGKαζ deficiency in Tregs led to enhanced Tfh and GC-B cell as well as non-GC B cell responses with prominent polarization to the Tfh2/13 lineage and IgG1/IgE responses, which provided mechanistic explanation of development of autoantibodies and lupus-like diseases and the IgG1-predominant autoimmunity in Treg-αζDKO mice. The increased Tfh and GC-B cells could be caused by a potential impaired Treg/Tfr-cell-mediated suppressive mechanism yet to be defined, by increased Treg/Tfr-cells to exTreg-Tfh cell conversion (as will be described later), by the inflammatory environment of dysregulated T helper cells and effector CD8 T cells, and/or by positive feedbacks or bystander activations between Tfh and GC B cells.

### Dysregulated Tfr-cells in Treg-αζDKO mice

Tfr-cells suppress GC responses by inhibiting Ig class-switch and Tfh-cell function, and their differentiation is Bcl6-dependent (Sage and Sharpe, 2016; Fu et al., 2018; Chung et al., 2011). In *Dgka^-/-^z^f/f^-Foxp3^YFPCre/YFPCre^* mice, Foxp3^+^CD4^+^CXCR5^+^PD-1^+^ Tfr-cells were increased (Figure 7—figure supplement 2A,B), at least as a result of enhanced proliferation but not survival (Figure 7—figure supplement 2C,D). Within CXCR5^+^PD-1^+^ CD4^+^T cells, the Foxp3^+^ Tfr to Foxp3^-^ Tfh cell ratios were increased (Figure 7—figure supplement 2E,F). αζDKO Tfr-cells upregulated CTLA-4, PD-1, CD69, and ICOS but downregulated CD25 and Nrp1 (Figure 7—figure supplement 2G,H). Mature Tfr cells downregulate CD25 to avoid IL-2 signal mediated inhibition (Ritvo et al., 2017; Kumar et al., 2021; Botta et al., 2017). Decreased CD25 in αζDKO Tregs and Tfr-cells could promote Tfr-cells differentiation, which might partially contribute to increased Tfr-cells. αζDKO Tfr-cells also upregulated Bcl6 (Figure 7—figure supplement 2I), accompanying increased GATA3^+^Bcl6^+^ Tfr2/13 cells and GATA3^-^Bcl6^+^ Tfr-cells (Figure 7—figure supplement 2I,J) and thus bias toward Tfr2/13 cells. Consistent with increased Tfr-cells, αζDKO Tregs were enriched in Tfr/Tfh-associated gene signatures (Figure 7—figure supplement 2K), characterized by upregulation of positive regulators (*Bcl6*, *Cxcr5*, *Pdcd1*, *Ascl2*, *Batf*, *Icos*, *Pou2af1*, *Cxcr5*, and *Sh2d1a* [encoding the adaptor molecule SAP]) of Tfh-cell and/or Tfr-cell differentiation. Although it is unclear whether αζDKO Tfr-cells were impaired in suppressive function, they gained expression of Tfh-associated cytokine *Il21* as well as Th2-associated cytokines *Il4* and *Il13*, which might have contributed to the IgG1/IgE-predominant antibodies and autoimmunity in Treg-αζDKO mice. Of note, some Tfh cells upregulate Foxp3 in GCs (Jacobsen et al., 2021). Of note, our data do not rule out that αζDKO Tfr-cells might contain some Tfh-derived Foxp3^+^ cells.

### Development of lupus-like diseases in female *Foxp3^YFPCre/+^* heterozygous *Dgka^-/-^z^f/f^-Foxp3^YFPCre/+^* mice

**Figure 8.**
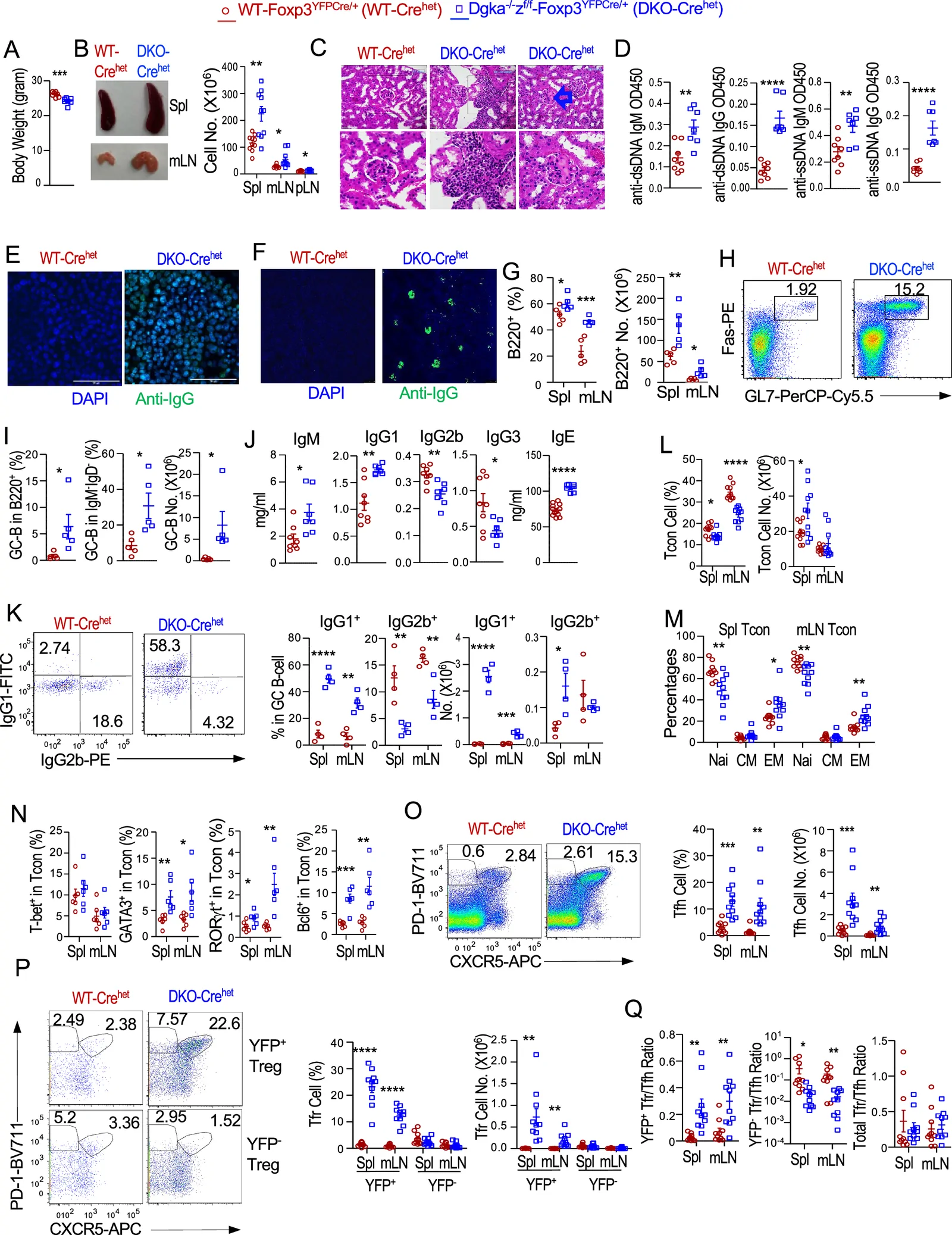
Development of autoimmune diseases and deregulated Tfh/Tfr cell and GC-responses in female *Dgka^-/-^z^f/f^-Foxp3^YFPCre/+^* mice. Three–nine-month-old female *Dgka^-/-^z^f/f^-Foxp3^YFPCre/+^* (DKO-Cre^het^) and WT*-Foxp3^YFPCre/+^* (WT-Cre^het^) mice were analyzed. (**A**) Body weights. (**B**) Representative picture of spleen and mLNs, total cell numbers in the indicated organs. (**C**) H&E staining of kidney thin sections. Bottom low shows higher magnification. (**D**) Serum anti-dsDNA and ssDNA autoantibodies. (**E**) Antinuclear antibodies. (**F**) IgG deposition in the kidney. (**G**) Total B cell percentages and numbers. (**H**) Fas and GL7 staining in splenic B220^+^ cells. (**I**) GC-B cell percentages in splenic B220^+^ and B220^+^IgM^-^IgD^-^ (DN) B cells and GC-B cell numbers in B220^+^ B cells. (**J**) Serum Ig levels. (**K**) IgG1^+^ and IgG2b^+^ cells in GC-B cells. (**L**) CD4^+^Foxp3^-^ Tcon percentages and numbers. (**M**) Naïve and effector cell percentages in Tcons. (**N**) Percentages of T-bet^+^, GATA3^+^, RORγt^+^, and Bcl6^+^ cells in Tcons. (**O**) Assessment of Tfh cells. Representative FACS plots show gating of CXCR5^+^PD-1^+^ Tfh and CXCR5^-^PD-1^+^ Tph cells in splenic Tcons. Scatter plots show Tfh percentages and numbers. (**P**) Assessment of Tfr cells. Representative FACS plots show gating of CXCR5^+^PD-1^+^ Tfr cells in splenic YFP^+^ and YFP^-^ Foxp3^+^ Tregs. Scatter plots show Tfr percentages and numbers. (**Q**) Scatter plots show Tfr/Tfh cell ratios. Data shown are representative of or pooled from 5 to 10 experiments except four experiments for **K**. *, p<0.05; **, p<0.01; ***, p<0.001; ****, p<0.0001 determined by two-tailed pair-wise (**A**, data with lines connecting WT-Cre^het^ and αζDKO-Cre^het^ mice) or unpaired Student *t* test.

Female *Dgka^-/-^z^f/f^-Foxp3^YFPCre/+^* (DKO-Cre^het^) mice contained both *Dgka^-/-^* control (called WT for simplicity) and αζDKO-Tregs. Surprisingly, they also lost weight and manifested lymphoproliferative/autoimmune disorders, albeit less severely than *Dgka^-/-^z^f/f^-Foxp3^YFPCre/YFPCre^* mice (Figure 8A-F, Figure 8—figure supplement 1A). They had increased B-cells (Figure 8G); GC B-cells within both total B220^+^ and B220^+^IgM^-^IgD^-^ DN B-cells (Figure 8H and I); serum IgM, IgG1, and IgE (Figure 8J); and IgG1^+^ in GC B-cells (Figure 8K) but decreased serum IgG2b and IgG3 and IgG2b^+^ cells. The increased CD23 in B-cells further supported elevated serum IgE (Figure 8—figure supplement 1B). In DKO-Cre^het^ mice, CD4^+^Foxp3^-^ Tcons were decreased in percentages but not in numbers owing to disproportional increases of B-cells (Figure 8L). They contained increased effector but decreased naïve T-cells (Figure 8M) accompanying increased percentages of GATA3^+^, RORγt^+^, and Bcl6^+^ but not T-bet^+^ CD4^+^Foxp3^-^ Tcons (Figure 8N). They had increased Tfh-cells (Figure 8O) with elevated PD-1, ICOS, and CXCR5 (Figure 8—figure supplement 1C). In DKO-Cre^het^ mice, YFP^+^ but not YFP^-^ Tfr-cells were increased (Figure 8P). The YFP^+^Tfr/Tfh cell ratios were increased but YFP^-^/Tfh cell ratios were decreased, although total Tfr/Tfh cell ratios were not obviously altered (Figure 8Q). Together, these data suggested that DGKα and ζ intrinsically inhibited Tfr-cell differentiation and that αζDKO Tregs/Tfr-cells and/or exTregs gained dominant functions that could overpower WT Treg/Tfr-cells to cause autoimmune diseases.

**Figure 8—figure supplement 1.**
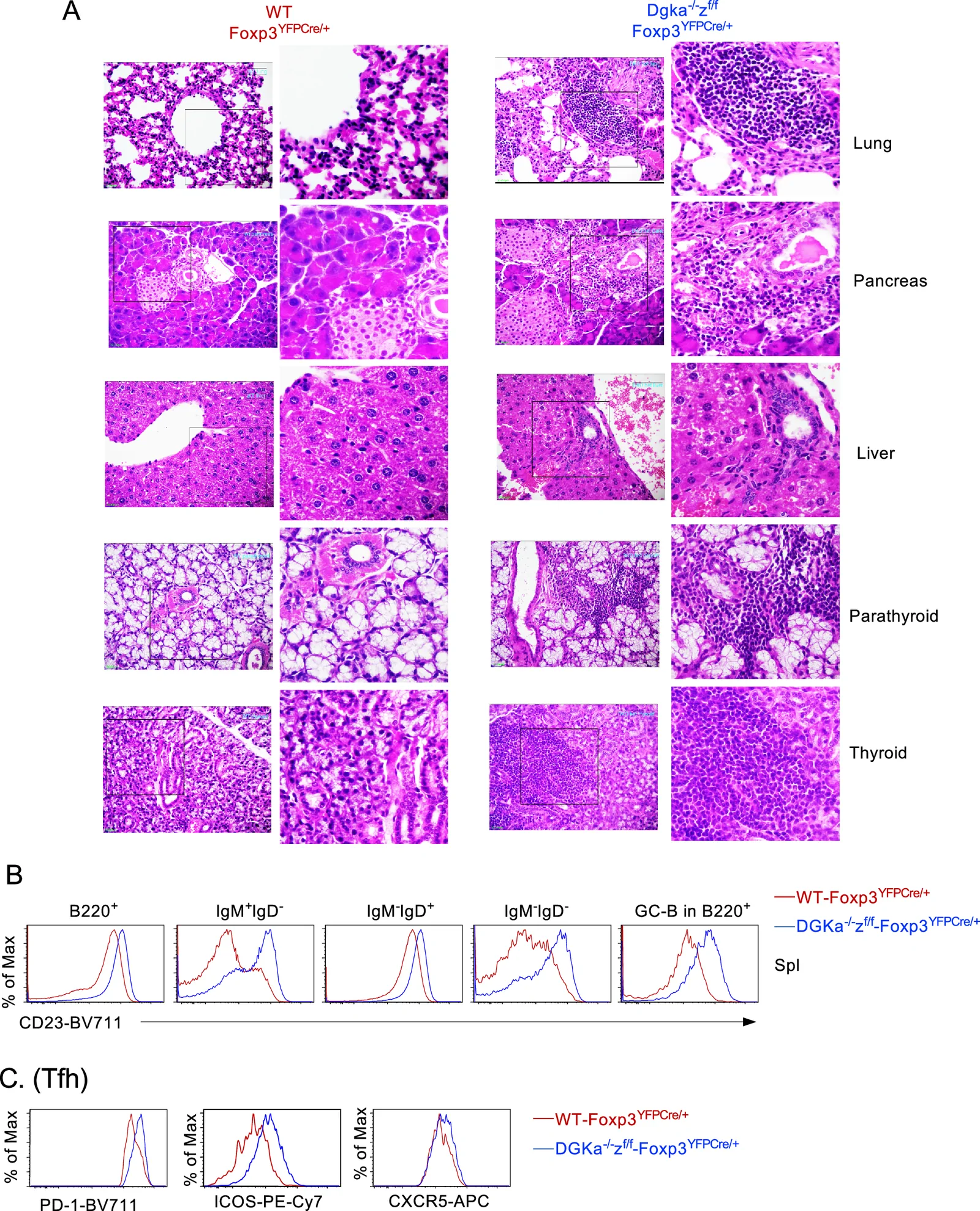
Development of autoimmune diseases in female *Dgka^-/-^z^f/f^-Foxp3^YFPCre/+^* mice. Three- to nine-month-old female *Dgka^-/-^z^f/f^-Foxp3^YFPCre/+^* (DKO-Crehet) and *WT-Foxp3^YFPCre/+^* (WT-Crehet) mice were analyzed. (**A**) Representative H&E staining of thin sections of the indicated organs. (**B**) CD23 levels in the indicated B cell populations. (**C**) PD-1, ICOS, and CXCR5 levels in Tfh cells.

### DGKαζ deficiency conferred CD28-independent Treg, Tfr-cell, and exTreg-Tfh-cell development and homeostasis and dysregulated GC responses and autoimmune diseases

**Figure 9.**
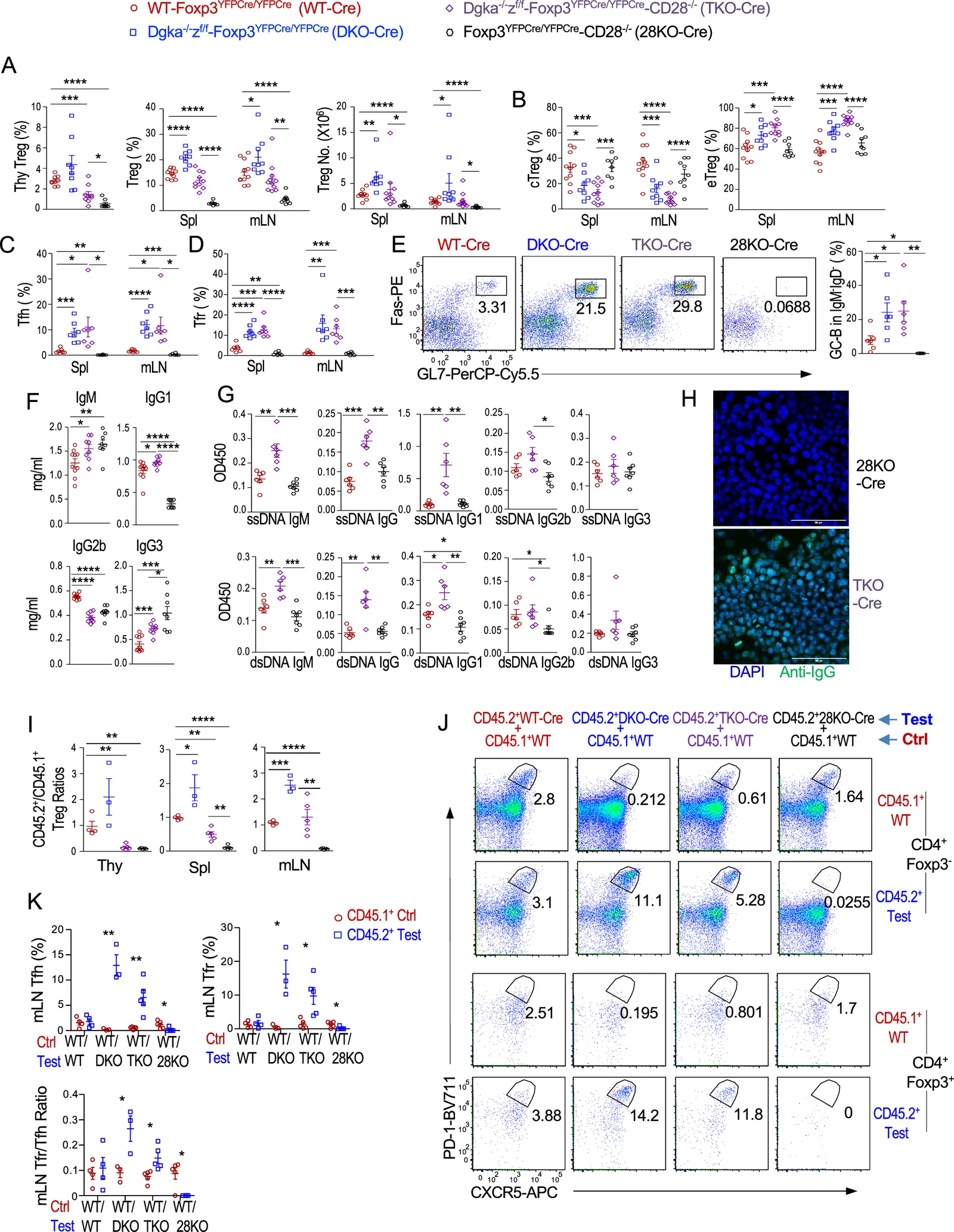
Treg-specific DGKαζ deficiency conferred CD28-independent Treg development/homeostasis and GC responses and accelerated Treg-to-exTreg/exTreg-Tfh conversion. (**A–H**) Analyses of WT*-Foxp3^YFPCre/YFPCre^*, *Dgka^-/-^z^f/f^-Foxp3^YFPCre/YFPCre^* (DKO), *Dgka^-/-^z^f/f^-Foxp3^YFPCre/YFPCre^-CD28^-/-^* (TKO), and *Foxp3^YFPCre/YFPCre^-CD28^-/-^* (CD28KO). (**A**) Treg percentages and numbers (mean ± SEM) in the thymus, spleen, and mLNs. (**B**) Mean ± SEM of cTreg and eTreg percentages. (**C**) Tfh-cell percentages. (**D**) Tfr-cell percentages. (**E**) Representative FACS plots showing Fas and GL7 staining in live gated splenic B220^+^IgM^-^IgD^-^ (DN) B cells. Scatter plots show mean ± SEM of GC-B cell percentages. (**F**) Serum antibody levels. (**G**) Serum anti-ssDNA and dsDNA autoantibody levels. (**H**) Serum antinuclear antibodies in *CD28^-/-^* and TKO mice. (**I–K**) Analyses of mixed BM chimeric mice reconstituted with a mixture of BM cells of CD45.1^+^ WT BM cells with either CD45.2^+^ WT*-Foxp3^YFPCre/YFPCre^*, *Dgka^-/-^z^f/f^-Foxp3^YFPCre/YFPCre^* (DKO), *Dgka^-/-^z^f/f^-Foxp3^YFPCre/YFPCre^-CD28^-/-^* (TKO), or *Foxp3^YFPCre/YFPCre^-CD28^-/-^* (28KO) BM cells. (**I**) Ratios of CD45.2^+^ test Treg percentages in CD4^+^ T cells/CD45.1^+^ WT Treg percentage in CD4^+^ T cells in individual chimeric mice. (**J**) Representative FACS plots showing PD-1 and CXCR5 staining in live gated mLN CD45.1^+^ control and CD45.2^+^ test CD4^+^Foxp3^-^ Tcons and CD4^+^Foxp3^+^ Tregs. (**K**) Percentages of Tfh and Tfr cells as well as Tfr/Tfh ratios of CD45.1^+^ and CD45.2^+^ origins in individual chimeric mice. Data shown are representative of or pooled from 6 to 11 experiments for A–H and 3–5 experiments for I–K. *, p<0.05; **, p<0.01; ***, p<0.001; ****, p<0.0001 by two-tailed unpaired Student t-test.

CD28 costimulatory signal promotes Ras-Erk1/2 and PI3K/mTOR signaling and is critical for Treg and Tfr development and homeostasis and for Tfh differentiation to promote GC responses and humoral immunity (Walker et al., 1999; Tai et al., 2005; Zhang et al., 2017). Enhanced mTORC1/2 and Erk1/2 activation in αζDKO-Tregs prompted us to examine whether Treg-αζDKO relieved their dependence on CD28. *Foxp3^YFPCre/YFPCre^-CD28^-/-^* (28KO-Cre) mice had very few Tregs; *Dgka^-/-^z^f/f^-Foxp3^YFPCre/YFPCre^-CD28^-/-^* (TKO-Cre) Tregs had increased slightly in the thymus and obviously in the periphery (Figure 9A), accompanying increased e/cTreg ratios (Figure 9B).

**Figure 9—figure supplement 1.**
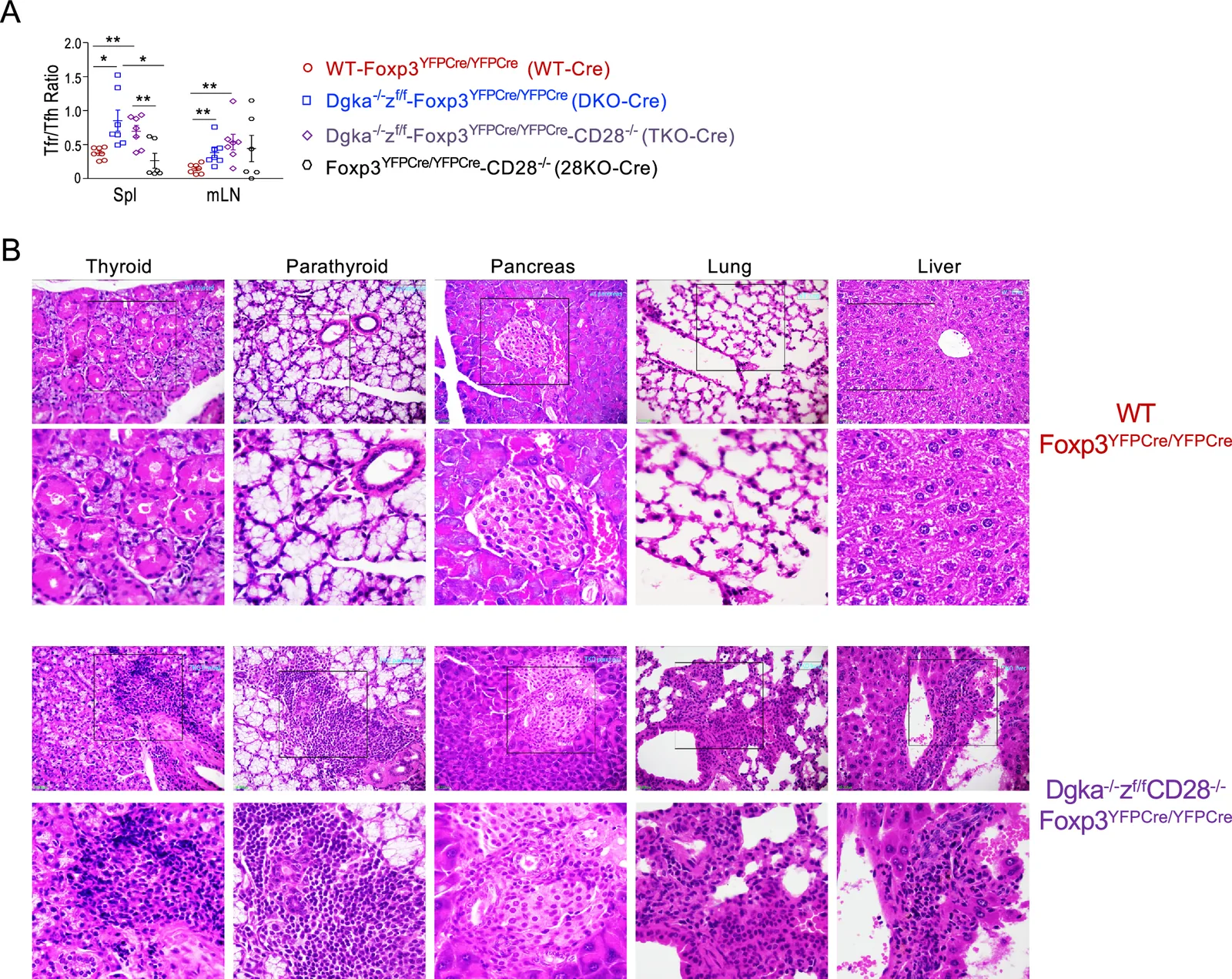
Altered Tfr/Tfh cell ratios and development of autoimmune diseases in *Dgka^-/-^z^f/f^-Foxp3^YFPCre/YFPCre^*-CD28^-/-^ mice. (**A**) Tfr/Tfh cell ratios in the indicated mice. (**B**) Representative H&E staining of tissue thin sections in the indicated mice are shown. Data shown are pooled from 5 to 10 experiments. *, p<0.05; **, p<0.01 determined by two-tailed unpaired Student t-test.

CD28KO-Cre mice were deficient in Tfh/Tfr-cells (Figure 9C and D) and GC B-cells (Figure 9E). They contained elevated serum IgM and IgG3 but reduced IgG1 and IgG2b (Figure 9F). However, most of these phenotypes, with the exception of IgG2b, were reversed in TKO-Cre mice. TKO-Cre mice even had more Tfh, Tfr, and GC B-cells as well as increased Tfr/Tfh cell ratios than WT mice (Figure 9C-E, Figure 9—figure supplement 1A). Similar to αζDKO-Cre mice, TKO-Cre mice also developed IgG1-dominant autoantibodies and multiorgan autoimmune diseases (Figure 9G, H, Figure 9—figure supplement 1B). Thus, Treg-αζDKO not only completely or partially reversed defects in Tregs, Tfr, and GC B-cells and humoral immunity caused by CD28 deficiency but also triggered IgG1-predominant autoimmunity independent of CD28.

In mixed BM chimeric mice reconstituted with a mixture of BM cells of CD45.1^+^ WT BM cells with either CD45.2^+^ WT-Cre, αζDKO-Cre, TKO-Cre, or 28KO-Cre BM cells, TKO-Cre Tregs were also increased, especially in the spleen and LNs (Figure 9I). Thus, DGKαζ activities intrinsically enforce CD28 dependence for Treg development/homeostasis and cTreg-to-eTreg differentiation.

Highly interestingly, although virtually no Tfr and Tfh-cells developed from 28KO-Cre BM cells, TKO-Cre-derived CD4^+^Foxp3^+^ Tregs and CD4^+^YFP^-^Foxp3^-^ Tcons contained increased Tfr and Tfh-cells even compared with WT-Cre controls in the mixed BM chimeric mice (Figure 9J–K). The Tfr/Tfh ratios were also increased within TKO-derived CD4^+^ T cells (Figure 9K). Because the canonical TKO-Cre BM cell-derived CD4^+^Foxp3YFP^-^ Tcons were CD28 deficient but expressed WT DGKζ, and should be defective in Tfh-/Tfr-cell differentiation, these TKO-derived Tfh-cells should have converted from Tregs/Tfr-cells and deficient of DGKζ as well as DGKα, and αζDKO in Tregs should have conferred CD28-independent Tfr/Tfh differentiation. However, although it is unlikely, these data do not rule out that DGKα deficiency in Tcons due to germline deficiency in the context of DGKαζ double deficiency in Tregs could play a role in promoting Tfh cell differentiation.

### Accelerated conversion of αζDKO Treg to exTregs and exTreg-Tfh cells and pathogenicity of αζDKO Tregs/exTregs in IgG1-predominant GC B-cell responses and autoimmunity

**Figure 10.**
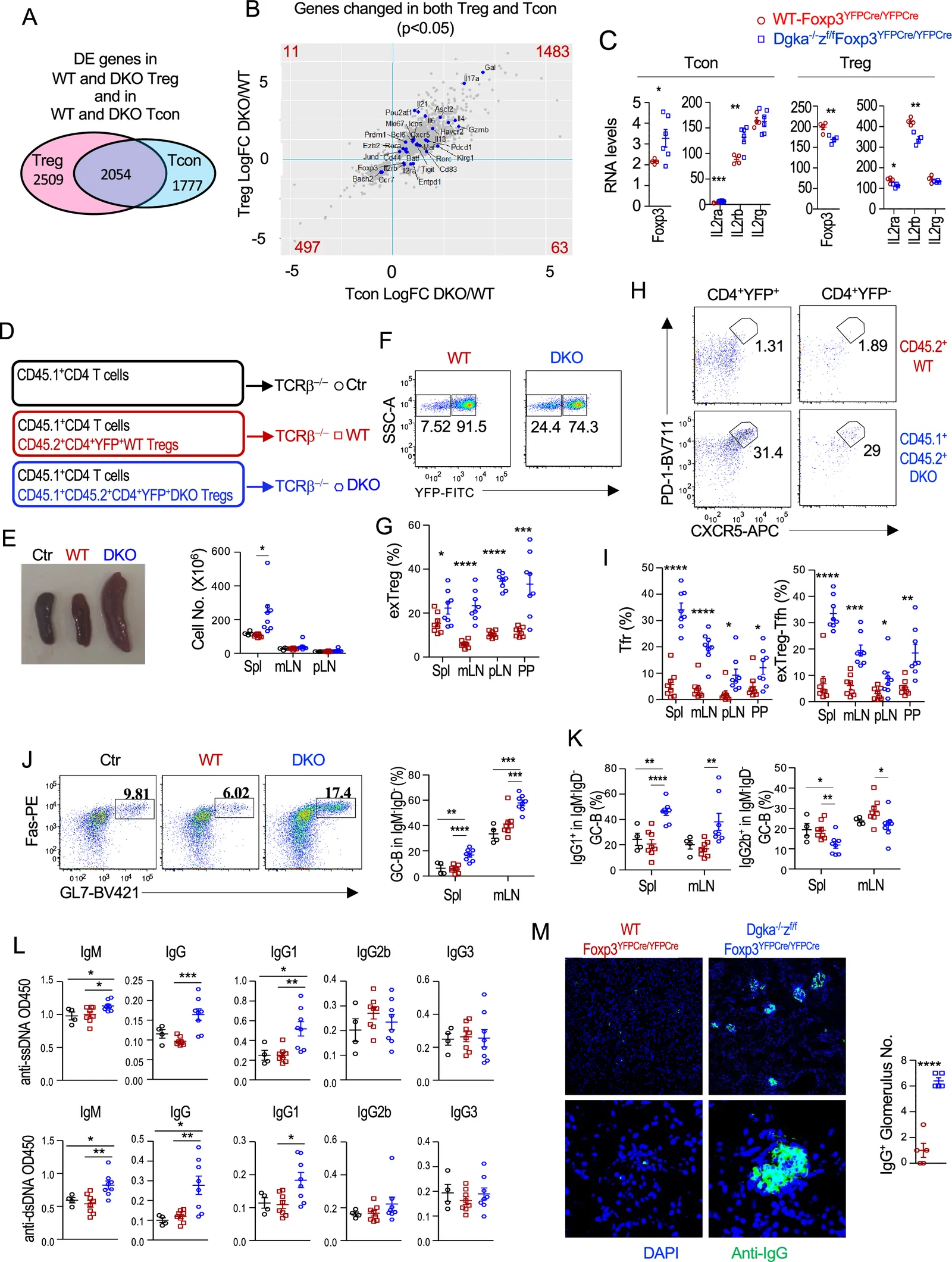
Enhanced conversion to exTreg/exTreg-Tfh cells and pathogenicity of αζDKO Tregs. (**A–C**) Comparison of DEGs in both Treg-αζDKO Tregs and Tcons compared with their corresponding WT controls. (**A**) DEGs that were shared or not shared in αζDKO Tregs and Tcons. (**B**) Concordant and discordant expression of DEGs in Treg-αζDKO Tregs and Tcons. (**C**) Expression of indicated Treg-associated genes in Treg-αζDKO Tregs and Tcons. (**D–M**) Adoptive transfer experiments. 5X10^5^ double-sorted CD45.2^+^ Tregs from WT*-Foxp3^YFPCre/YFPCre^* or CD45.1^+^CD45.2^+^ Tregs from *DGKα^-/-^ζ^f/f^-Foxp3^YFPCre/YFPCre^* mice were co-injected *i.v*. with 1X10^6^ WT CD45.1^+^ CD4^+^ T cells into TCRβ^-/-^ mice. Serum was collected and mice were euthanized for the experiment 10 weeks after transfer. (**D**) Experimental scheme. 5 X 10^5^ double-sorted CD45.2^+^ Tregs from WT*-Foxp3^YFPCre/YFPCre^* or CD45.1^+^CD45.2^+^ Tregs from *Dgka^-/-^z^f/f^-Foxp3^YFPCre/YFPCre^* mice were co-injected *i.v*. with 1X10^6^ WT CD45.1^+^ CD4^+^ T cells into TCRβ^-/-^ mice. Serum was collected and mice were euthanized for the experiment 10 weeks after transfer. (**E**) Spleen sizes and total cell numbers (mean ± SEM). (**F**) Representative FACS plot showing YFP levels in live gated CD45.2^+^ or CD45.1^+^CD45.2^+^CD4^+^TCRβ^+^ cells. (**G**) Scatter plot showing means ± SEM of CD4^+^YFP^-^ exTreg percentages. (**H**) Representative FACS plots showing PD-1 and CXCR5 staining in YFP^+^ 45.2^+^/CD45.1^+^CD45.2^+^CD4^+^TCRβ^+^ Tregs and YFP^-^ CD45.2^+^ or CD45.1^+^CD45.2^+^CD4^+^TCRβ^+^ exTregs. (**I**) Mean ± SEM of Tfr and ExTreg-Tfh cell percentages. (**J**) FACS plots showing Fas and GL7 staining in B220^+^ IgM^-^IgD^-^ B cells. Scatter plot showing mean ± SEM of GC-B cell percentages. (**K**) IgG1^+^ and IgG2b^+^ cells in GC-B cells in the spleen and mLNs. (**L**) Serial anti-ssDNA and -dsDNA antibody titers. (**M**) IgG deposition in the kidney. Representative immunofluorescence of anti-IgG staining of kidney sections is shown. Scatter plot shows mean ± SEM of IgG^+^ glomerulus numbers per 10X10 field. (**L**) is from one experiment and represents two experiments. Other data are representative of or pooled from four to eight experiments. *, p<0.05; **, p<0.01; ***, p<0.001; ****, p<0.0001 by two-tailed unpaired Student t-test.

ExTregs can be pathogenic owing to their expression of TCRs with relatively high affinities to self-antigens. If increased conversion of Tregs to exTregs occurred in *Dgka^-/-^z^f/f^-Foxp3^YFPCre/YFPCre^* mice, it should blend the CD4^+^Foxp3YFP^-^ T-cell population with exTregs that might express residue Treg signature genes. Among the 3831 and 4563 DEGs in *Dgka^-/-^z^f/f^-Foxp3^YFPCre/YFPCre^* CD4^+^YFP^-^ Tcons and CD4^+^YFP^+^ Tregs compared with their WT controls, 2054 of them were differentially expressed in both Tregs and Tcons (Figure 10A, Supplementary file 5). Within these 2054 genes, 1483 and 497 genes were concordantly increased or decreased in both Treg-αζDKO Tregs and Tcons, respectively (Figure 10B). Eleven genes with unclear functions in Tregs were increased in αζDKO Tregs but decreased in αζDKO Tcons. Sixty-three genes, including several Treg signature genes *Il2ra*, *Il2rb*, and, particularly, *Foxp3* were discordantly increased in Treg-αζDKO Tcons but decreased in Treg-αζDKO Tregs (Figure 10C). These data suggested that some Treg-αζDKO Tcons contained residual Treg-associated genes and were likely blended with exTregs. Consistent with this notion, we detected Cre-mediated recombination of *Dgkz^f/f^* alleles in sorted CD4^+^Foxp3YFP^-^Tcons from *Dgka^-/-^z^f/f^-Foxp3^YFPCre/YFPCre^* mice and female *Dgka^-/-^z^f/f^-Foxp3^YFPCre/+^* mice but not from *Dgka^+/+^zf^f/f^-Foxp3^YFPCre/YFPCre^* mice (Figure 10—figure supplement 1A), suggesting increased Treg-to-exTreg conversion in Treg-specific DGKα and ζ double but not DGKζ single deficient mice.

**Figure 10—figure supplement 1.**
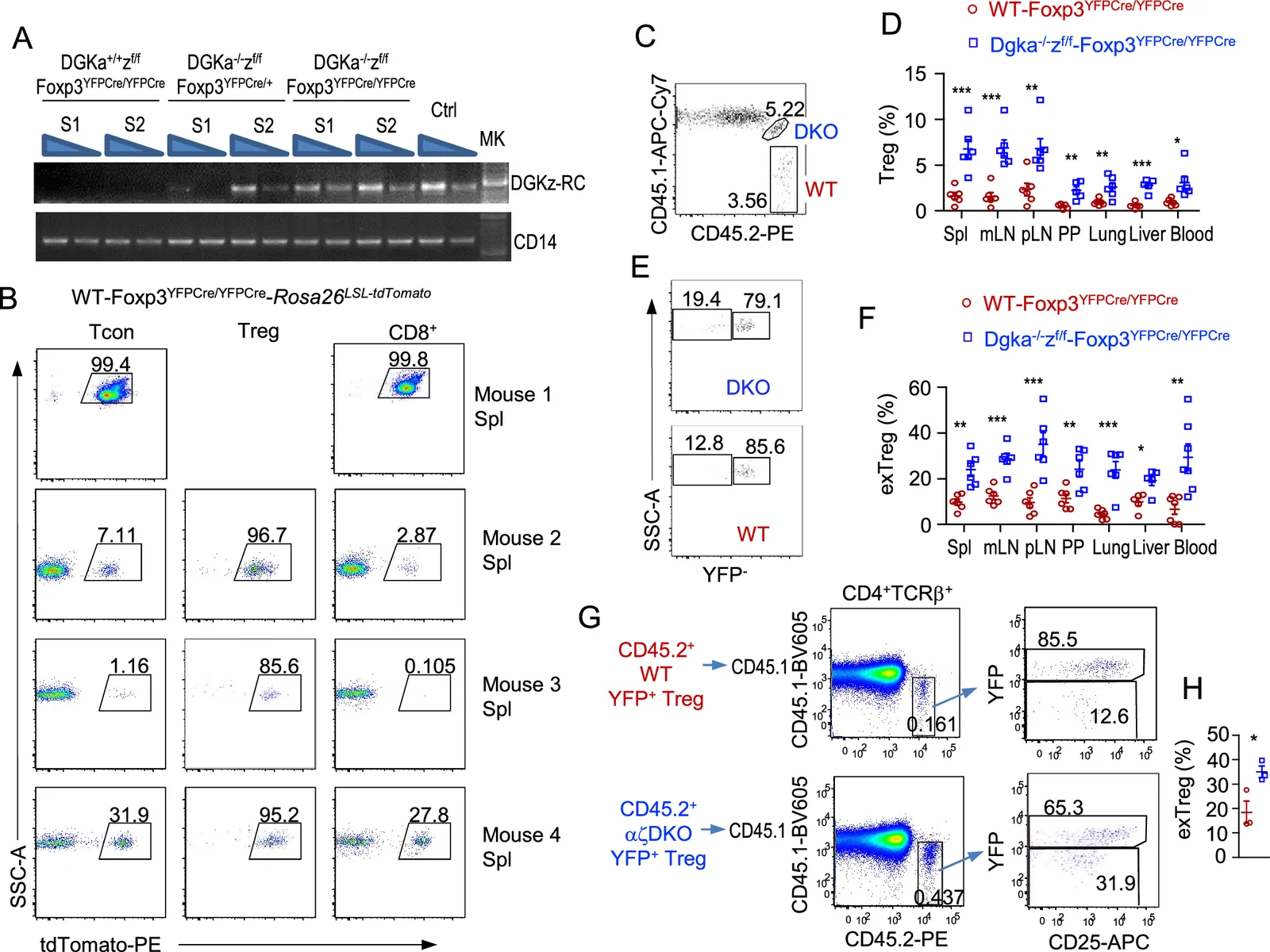
Enhanced αζDKO Treg conversion to exTregs/exTreg-Tfh cells. (**A**) Detection of Cre-mediated recombination in CD4+Foxp3YFP- Tcons in *Dgka^-/-^z^f/f^-Foxp3^YFPCre/YFPCre^* and *Dgka^-/-^z^f/f^-Foxp3^YFPCre/+^* mice but not in *Dgka^+/+^zf^f/f^-Foxp3^YFPCre/YFPCre^* mice. Ctrl: DNA from total LN cells (including both Tregs and Tcons) of *Dgka^-/-^z^f/f^-Foxp3^YFPCre/YFPCre^*. MK: DNA ladder. S1 and S2 indicate different mice. One of two experiments of total four *Dgka^+/+^zf^f/f^-Foxp3^YFPCre/YFPCre^* YFPCre mice and four αζDKO mice is shown. The Cre-mediated recombination of floxed *Dgkz* allele was detected with two *Dgkz* primers (forward: 5′-GGCACACTGGAGACTCTCAC-3′ and reverse: 5′-CTAGCTTGGGCTGCAGGT-3′). The *Cd14* genomic DNA was amplified using the following primers: Cd14forward: 5′-GCTCAAACTTTCAGAATCTACCGAC-3′ and Cd14reverse: 5′-AGTCAGTTCGTGGAGGCCGGAAATC-3′. (**B**) Variegated tdTomato expression in *Foxp3^YFPCre/YFPCre^-Rosa26^LSL-tdTomato^* mice. (**C–F**) TCRβ^-/-^ mice were coinjected with a mixture of CD45.1^+^CD45.2^-^ WT CD4^+^ T cells, CD45.1^-^CD45.2^+^ CD4^+^YFP^+^ WT Tregs, and CD45.1^+^CD45.2^+^ CD4^+^YFP^+^ Treg-αζDKO Tregs and analyzed 8 weeks later. (**C**) Representative FACS plots showing CD45.1 and CD45.2 staining in live gated mLN CD4^+^TCRβ^+^ cells. (**D**) Percentages of WT and αζDKO Treg-derived cells within adoptively transferred CD4^+^TCRβ^+^ cells. (**E**) FACS plots showing YFP levels in live gated cells derived from WT or αζDKO Tregs. (**F**) Percentages of YFP- exTregs. Data shown are representative or pooled from five to seven experiments. *, p<0.05; **, p<0.01; ***, p<0.001 determined by two-tail unpaired Student t-test. (**G, H**) 5X10^5^ Foxp3YFP^+^ Tregs from *Dgka^-/-^z^f/f^-Foxp3^YFPCre/YFPCre^* and *WT-Foxp3^YFPCre/YFPCre^* mice were separately i.v. injected into CD45.1^+^ C57BL6/J mice 4 hr after irradiation (400 Rad). LNs from recipient mice were examined 14 days later. (**G**) Representative FACS plots showing CD4^+^TCRβ^+^ T cells. (**H**) Scatter plots show mean ± SEM of data pooled from three experiments. *, p<0.05 determined by two-tail unpaired Student t-test. Figure 10—figure supplement 1—source data 1.Original agarose gel image of Cre-mediated Dgkzf/f recombination. Figure 10—figure supplement 1—source data 2.Original agarose gel image of Cre-mediated Dgkzf/f recombination with label.

We initially used *Foxp3^YFPCre/YFPCre^-Rosa26^LSL-tdTomato^* reporter mice, an approach that has been used to map Treg fate, to further assess Treg to Tcon conversion. However, most *Foxp3^YFPCre/YFPCre^-Rosa26^LSL-tdTomato^* mice displayed variegated expression of tdTomato in many different cell types including CD8^+^ T-cells, B cells, CD4^+^Foxp3^-^ Tcons, and other immune cells in addition to Tregs among different mice (Figure 10—figure supplement 1B). In extreme cases, the entire mouse could turn reddish, suggesting Cre-mediated germline recombination.

To further examine Treg-to-exTregs/exTreg-Tfh-cell conversion and to assess the pathogenicity of αζDKO Tregs/exTregs, we co-injected CD45.1^+^WT CD4^+^ T-cells (containing both Tregs and Tcons) with double-sorted CD4^+^YFP^+^TCRβ^+^ Tregs from either CD45.2^+^WT-*Foxp3^YFPCre/YFPCre^* or CD45.1^+^CD45.2^+^*Dgka^-/-^z^f/f^-Foxp3^YFPCre/YFPCre^* mice (both >99% purity) into TCRβ^-/-^ mice (Figure 10D). Two to three months after transfer, recipients of αζDKO-Tregs manifested splenomegaly (Figure 10E), increased YFP^-^ exTregs within the transferred CD45.2^+^CD4^+^TCRβ^+^ population (Figure 10F and G), increased CXCR5^+^PD-1^+^ Tfr-cells within the YFP^+^ population, increased CXCR5^+^PD-1^+^ exTreg-Tfh-cells within the CD45.2^+^CD4^+^TCRβ^+^YFP^-^ exTregs (Figure 10H and I), and increased GC B-cells and IgG1^+^ cells within GC B-cells (Figure 10J and K). Moreover, αζDKO-Treg but not WT-Treg recipients developed elevated IgM- and IgG1-predominant autoantibodies (Figure 10L) and glomerular IgG deposition (Figure 10M). Increased αζDKO-Treg-to-exTreg conversion was also observed when both WT-Tregs and αζDKO-Tregs were co-injected into the same TCRβ^-/-^ hosts and thus in the same environment (Figure 10—figure supplement 1C-F) or when they were injected individually into mildly irradiated WT hosts (Figure 10—figure supplement 1G,H). Together, these data provided strong evidence that DGKαζ double deficiency accelerated Treg/Tfr-cell conversion to exTregs and exTreg-Tfh-cells. These data also suggested that DGKαζ deficient exTreg/exTreg-Tfh-cells may trigger deregulated GC B-cell and IgG1-predominant autoantibody responses. Of note, WT and αζDKO Tregs contained different ratios of effector Treg lineages and Tfr cells, which could partially contribute to the phenotypes observed. Future adoptive transfer experiments with individual Treg sublineages should provide additional insight into the mechanistic control of Treg to exTreg/Tfh cell conversion.

## Discussion

We have demonstrated that DGKαζ is critical for Tregs to maintain normal homeostasis and phenotypic and functional properties. In the absence of DGKαζ, Treg/Tcon ratios in lymphoid organs are reset to high levels due to enhanced Treg homeostatic proliferation; cTreg differentiation to eTreg is enhanced; and both cTregs and/or eTregs express abnormal levels of signature molecules. Increased CTLA-4, TIGIT, and ICOS may contribute to enhanced in vitro contact inhibition; increased PD-1 may reflect enhanced Treg activation and entry of functional exhaustion; decreased CD25 may destabilize Tregs (Chen et al., 2012; Joller et al., 2014; Toomer et al., 2019; Tan et al., 2021). The abnormalities in Tregs and the development of multiorgan autoimmune diseases and increased effector CD8^+^ T-cells in Treg-αζDKO mice but not in DGKα or ζ single knockout mice suggest that DGKα and ζ synergistically prevent Tregs from functional impairment in vivo. However, due to the lack of a Treg-specific DGKα-deficient mouse model and the fact that Treg-αζDKO mice are DGKα germline deficiency, we could not completely rule out that DGKα deficiency in Tcons, CD8^+^ T cells, B cells, and/or other cells might contribute to the autoimmune phenotypes in the context of DGKαζ double deficiency in Tregs.

Tregs differentiate to multiple effector sublineages such as Treg1, Treg2, Treg17, and Tfr sublineages. However, proinflammatory cytokines and functions associated with Th-cells are usually suppressed in WT-Treg sublineages. In Treg-αζDKO mice, Treg2, Treg17, and Tfr-cells but not Treg1 cells are increased in numbers. At present, we do not know if the increases of Treg effector cells would enhance their pertinent suppressive functions. However, αζDKO Tregs express elevated Th-associated cytokines such as IFNγ, IL-17, and IL-4, suggesting that DGKαζ serves as a signal checkpoint to prevent Treg effector lineages differentiation and may also inhibit expression of cytokines that are usually associated with Th cells in Tregs. While we suspect that elevated proinflammatory cytokine expression by αζDKOTregs/exTregs may contribute to autoimmunity in Treg-αζDKO mice, further studies are needed to make firm conclusions.

Although Treg instability can lead to autoimmune diseases because of their self-reactivity (Zhou et al., 2009; Bailey-Bucktrout et al., 2013; Komatsu et al., 2014), WT Treg-to-exTreg conversion is normally very limited in the steady state (Hori, 2014; Sakaguchi et al., 2013; Levine et al., 2017; Miyao et al., 2012; Rubtsov et al., 2010). Treg-to-exTreg-Tfh cell conversion has been rarely reported (Tsuji et al., 2009). A recent study identified that a CD25^low^ Tfr-cell population tends to lose function and become ‘ex-Tfr’ cells (Hou et al., 2019). Mechanisms that ensure Treg stability are still not fully understood. Regulation of exTreg-Tfh-cell generation, functions, and pathogenicity is virtually unknown. We have revealed accelerated conversion of αζDKO-Tregs to exTregs, especially exTreg-Tfh-cells, and expansion of these cells in a CD28-independent manner. Upregulated ICOS may equip αζDKO exTreg-Tfh-cells with enhanced capability to receive positive-feedback signals from already increased GC B-cells to further promote expansion and function of exTreg-Tfh-cells. These αζDKO Tregs/exTreg cells are capable of triggering IgG1-predominant autoimmunity and GC B-cells. Our data indicate that normal Treg stability requires synergistic function of DGKα and ζ as DGKαζ activity serves as a signal checkpoint by braking Treg-to-exTreg/exTreg-Tfh cell conversion and by limiting numbers and functions of pathogenic IL4/13/21-expressing Tregs and exTreg/exTreg-Tfh cells to prevent IgG1-/IgE-predominant GC B-cell responses and/or autoimmunity.

DGKαζ double deficiency could affect Treg stability via multiple mechanisms. In αζDKO-Tregs, mammalian sterile 20-like kinase 1 (Mst1; Li et al., 2015), TET1/2/3 (Yue et al., 2016), and ring finger protein 31 (RNF31; Luo et al., 2016), which promote Treg stability, were decreased; Pim-2 Kinase (Deng et al., 2015), cyclin-dependent kinase 2 (CDK2; Morawski et al., 2013), and tumor progression locus 2 (Tpl2; Li et al., 2016), which decrease Foxp3 and Treg stability, are increased (Supplementary file 1). Foxo1 is important for Treg homeostasis and stability but inhibits Tfh- and Th17-cell differentiation (Kerdiles et al., 2010); reduced Foxo activity may destabilize αζDKO-Tregs and promote their exTreg differentiation. CD25 is important for Treg stability and is downregulated in Tfr-cells, which appear less stable than other Tregs (Toomer et al., 2019; Hou et al., 2019). IL-2 signal also inhibits Tfh cell differentiation (Ballesteros-Tato et al., 2012). Decreased CD25 in αζDKO-Tregs may destabilize them and accelerate their conversion to exTreg cells. Additionally, αζDKO-Tregs have altered metabolism such as enhanced glycolysis, are hyperproliferative, and upregulate many Th-/Tfh-associated TFs. Whether these abnormalities contribute to Treg destabilization remains to be investigated.

Signals from the TCR and CD28 are important for Treg maintenance and function (Delpoux et al., 2014; Levine et al., 2014; Zhang et al., 2017). How these signals are regulated is not fully understood. We have revealed that DGKα and ζ impose Treg’s reliance on CD28 costimulatory signal. Treg homeostasis, Tfh-/Tfr-cell differentiation and function, and GC responses depend on CD28 (Walker et al., 1999; Tai et al., 2005; Zhang et al., 2017). CD28 deficiency causes severely decreased Tregs; virtual absence of Tfh, Tfr, and GC B-cells; and decreased serum IgG1 but increased IgG3 levels. αζDKO in Tregs/exTregs partially or completely reverses these phenotypes caused by CD28 deficiency. Using both transcriptomic analysis and intracellular staining, we showed that DGKαζ controls multiple DAG-mediated pathways downstream of the TCR to exert their roles in Tregs. Transcriptomic analyses reveal that αζDKO Tregs display altered signaling and metabolic and transcription programs such as TCR signaling, MAPK, NFκB, Foxo, cell cycle, chemokine, and many other pathways. Intracellular staining further confirms enhanced Erk1/2 and mTORC1/2 activation in αζDKO Tregs. Many of these pathways are important for Treg development, homeostasis, function, and/or stability. Enhanced DAG-Ras-Erk1/2 and mTOR signaling and enhanced uptake of glucose and glycolysis may trigger and fuel αζDKO-Treg proliferation, gain of proinflammatory function, and destabilization. Because both mTORC1 and mTORC2 are crucial for Tfh differentiation (Yang et al., 2016a; Zeng et al., 2016), enhanced mTOR activity in αζDKO-Tregs/exTregs could also intrinsically promote exTreg-Tfh differentiation/expansion, which might further promote Tfh differentiation via bystander activating mechanism as observed in Tsc1-deficient mice (Zhang et al., 2021). Future studies may illustrate how deregulation of each of these pathways may influence Treg stability and generation and function of exTreg-Tfh-cells as well as other aspects of Treg properties and functions.

In summary, we have demonstrated that DGKα and ζ play synergistic and critical roles in Tregs via tight control of multiple signaling pathways and metabolic and transcriptional programs. DGKα and ζ set normal Treg pool size by limiting their proliferation, restrain cTreg-to-eTreg differentiation, suppress Tregs’ proinflammatory programs, enforce Tregs’ dependence on CD28 costimulatory signal, and promote Tregs’ stability to brake Treg/Tfr cell-to-exTreg/exTreg-Tfh-cells conversion and subsequent expansion to prevent deregulated GC and autoantibody, especially IgG1/IgE predominant, responses. Our data provide the first evidence to suggest that exTreg-Tfh cells, at least in the Treg-αζDKO mouse model, are an important or even major source of pathogenic Tfh-cells that trigger deregulated autoantibody responses and autoimmune diseases. In line with many autoimmune diseases associated with deregulated Tfh and GC B-cells and elevated class-switched high-affinity autoantibodies, future studies should determine whether exTreg-Tfh-cells play important roles in other autoimmune diseases in both animal models and human patients.

## Methods

### Experimental animals

C57BL/6, B6-CD45.1^+^, B6-Thy1.1^+^, *Foxp3^YFPCre^*, *Rosa26^LSL-tdTomato^*, *Tcrb^-/-^* mice, *Rag1^-/-^*, and *Cd28^-/-^* mice were purchased from the Jackson Laboratory. *Dgka^-/-^* and *Dgkz^f/f^* mice were previously reported (Olenchock et al., 2006; Yang et al., 2016b). Mice were maintained in specific pathogen-free facilities at Duke University. Both male and female mice were used for experiments. Single cells from the indicated organs were resuspended in IMDM supplemented with 10% FBS, 1% penicillin/streptomycin, and 50 mM 2-mercaptoethanol (IMDM-10) according to standard protocols.

### Antibodies, reagents, and flow cytometry

Antibodies and reagents are listed in Supplementary file 6. Cells were stained with fluorescently conjugated antibodies in 2% FBS-PBS (PBS-2). Cell surface markers were stained at 4 °C for 30 min. For intracellular staining of TFs, signaling molecules such as phospho-S6, phospho-Akt Ser473, and phospho-Erk, and other molecules such as YFP, Ki67, and CTLA-4, cells were fixed and permeabilized utilizing the Foxp3/TF Staining buffer set. Intracellular staining for IgG1 and IgG2b was performed by using the BD Biosciences Cytofix/Cytoperm and Perm/Wash solutions. For cell death, Live/Dead Fixable Violet Dead Cell Stain (Invitrogen, Carlsbad, CA) was used according to the manufacturer’s protocol. Stained cells were acquired on a FACS Fortessa or Canto II (BD Biosciences) device. FACS data were analyzed with FlowJo software (Version 9.9.6).

### CD4 T-cell enrichment and sorting

To enrich CD4^+^ T-cells, total splenocytes and LN cells were resuspended in 500 μl of IMDM-10 and incubated with 50 μl of CD4 MicroBeads (Miltenyi Biotec, Cat. No. 130-117-043) at 4 °C for 30 min. After washing with IMDM-10, cells were resuspended in 2,000 μl PBS-2 and 1 mM EDTA. CD4^+^ T-cells were enriched using the LS columns (Miltenyi Biotec, Cat. No. 130-042-401). Enriched CD4^+^ cells were further stained with APC-anti-CD4, PE-Cy7-anti-CD8. Live CD4^+^CD8^-^YFP^+^ Tregs and CD4^+^CD8^-^YFP^-^ Tcons were sorted on an Aria II cell sorter (70 μm nozzle). Sorted cells were used to make RNA and DNA, or double sorted for injection.

### In vitro stimulation

Splenocytes and LN cells were stimulated with 50 ng/ml PMA and 500 ng/ml ionomycin in the presence of brefeldin A (1 ng/ml) for 4–5 hr. After cell surface staining, intracellular staining for Foxp3, GFP, IL-17A, IL-4, and IFN-γ was performed using the eBiosciences Foxp3 Cytofix/Cytoperm and Perm/Wash solutions. For Treg proliferation, 2x10^5^ CTV-labeled splenocytes were seeded in a U-bottom 96-well plate and stimulated with an anti-CD3 antibody (145–2 C11, 0.1 μg/ml) at 37 °C for 72 hr.

### Treg-mediated contact inhibition

CTV-labeled CD45.1^+^CD4^+^CD25^-^ WT Tcons (5x10^4^) were mixed with CD45.2^+^CD8^-^CD4^+^Foxp3YFP^+^ Tregs sorted from WT- or αζDKO-Foxp3^YFPCre/YFPCre^ mice at the ratios of 2:1 and 16:1 as well as 2x10^5^ mitomycin C-treated *Tcrb^-/-^* splenocytes and stimulated with an anti-CD3ε antibody (1 μg/ml). Cells were counted and stained for FACS analyses 72 hr later.

### BrdU incorporation assay

Mice were intraperitoneally injected with 1.5 mg of BrdU (150 μl of 10 mg/ml stock solution, MP Biomedicals, Cat No. 02100171-CF). Splenocytes and LN cells were stained for surface markers 8–10 hr after BrdU injection and then intracellular staining for BrdU according to the manufacturer’s protocol (BD Biosciences).

### Mixed BM chimeric mice

CD45.1^+^CD45.2^+^ WT mice were lethally irradiated (1000 rad) and intravenously injected with a mixture of BM cells (1.5–2×10^7^) from CD45.1^+^ WT and CD45.2^+^ WT*-Foxp3^YFPCre/YFPCre^*, *Dgka^-/-^z^f/f^-Foxp3^YFPCre/YFPCre^*, *Dgka^-/-^z^f/f^-Foxp3^YFPCre/YFPCre^-Cd28^-/-^*, or *Cd28^-/^-Foxp3^YFPCre/YFPCre^* mice. Chimeric mice were examined 8 weeks after irradiation.

### T cell adoptive transfer

CD45.2^+^ double-sorted WT or αζDKO Tregs (0.25×10^6^, purity >99%) were *i.p*. injected with 0.5×10^6^ enriched CD45.1^+^ WT CD4^+^ T cells into TCRβ^-/-^ mice and analyzed for 10–12 weeks after adoptive transfer (Figure 10D–M). For experiments in Figure 10—figure supplement 1C-F, a mixture of double-sorted 0.25×10^6^ CD45.2^+^ WT Tregs and CD45.1^+^CD45.2^+^ αζDKO Tregs as well as 0.5×10^6^ CD45.1^+^ CD4 T cells were co-injected into TCRβ^-/-^ mice. Recipient mice were analyzed 8 weeks later. For experiments in Figure 10—figure supplement 1G and H, 0.5 × 10^6^ CD45.2^+^ WT or αζDKO Tregs were injected into CD45.1^+^ WT mice 4 hr after mild irradiation (400 rad). Recipient mice were analyzed 14 days later.

### Glucose uptake assay

Two million splenocytes in 200 μl PBS from WT*-Foxp3^YFPCre/YFPCre^* and *Dgka^-/-^z^f/f^-Foxp3^YFPCre/YFPCre^* mice were seeded into a U-bottom 96-well plate in the presence or absence of 100 μM 2-(N-(7-Nitrobenz-2-oxa-1, 3-diazol-4-yl) Amino)–2-Deoxyglucose (2-NBDG; Life Technologies). After incubation at 37 °C with 5% CO_2_ for 30 min, 2-NBDG uptake was stopped by removing culture medium and washed with pre-chilled PBS twice. Cells were stained for surface markers before analysis with flow cytometry.

### Metabolic profiling

Sorted Tregs were washed with IMDM-10 and seeded in a 96-well plate at 2.5×10^5^ cells/well. Extracellular acidification rate (ECAR) and OCR were measured using an XFp Extracellular Flux Analyzer under glycolysis, mitochondrial stress, and mitochondrial fuel test conditions (Seahorse Bioscience/Agilent). For the glycolysis stress test, the assay buffer was made of nonbuffered DMEM medium supplemented with 2 mM glutamine, and d-glucose, oligomycin, and 2DG were sequentially injected at final concentrations of 10 mM, 1 µM, and 50 mM, respectively. For the mitochondrial stress test, the assay buffer was made of nonbuffered DMEM medium supplemented with 2.5 mM d-glucose, 2 mM glutamine, and 1 mM sodium pyruvate, and oligomycin, carbonyl cyanide 4-(trifluoromethoxy)phenylhydrazone, and rotenone/antimycin A were sequentially injected at final concentrations of 1 µM, 1 µM, and 500 nM, respectively. For the mitochondrial fuel test, the assay buffer was made of nonbuffered DMEM medium supplemented with 2.5 mM d-glucose, 2 mM glutamine, and 1 mM sodium pyruvate, and UK5099, etomoxir, and bis-2-(5-phenylacetamido-1,3,4-thiadiazol-2-yl)ethyl sulfide were sequentially injected at final concentrations of 2 µM, 4 µM, and 3 µM, respectively. Baseline ECAR (for the glycolysis stress test) and OCR (for the mitochondrial stress and mitochondrial fuel tests) values were averaged between technical replicates for these first three successive time intervals.

### Histology and IgG deposition in the kidney

Organs harvested from 6- to 16-month-old mice were fixed in 10% formalin overnight, preserved in 70% ethanol, and embedded in paraffin. The paraffin thin sections were stained with hematoxylin and eosin (H&E). Cryosections of kidneys frozen in OCT embedding medium were fixed with cold acetone for 5 min before rehydration in Tris-buffered saline (TBS, 50 mM Tris-Cl, pH 7.5, 150 mM NaCl). After being blocked with TBS containing 2% BSA and 5% normal donkey serum for 30 min, cryosections were incubated with an Alexa Fluor 488-anti-mouse IgG antibody at 4 °C overnight. Slides were washed for 15 min in TBS before mounting in Vectashield hard set with DAPI.

### Enzyme-linked immunosorbent assay and HEp-2 anti-nuclear antibody detection

Fifty microliters of appropriately diluted serum samples were added to 96-well plates precoated with anti-mouse Igκ and Igλ antibodies (2 mg/ml; Southern Biotech, Birmingham, AL) in 0.1 M carbonate buffer (pH 9.0). After incubation at 4 °C overnight and multiple washes, total and subtype Ig concentrations were detected using HRP-conjugated goat anti-mouse anti-IgM, IgG, IgG1, IgG2b, IgG3, and IgE antibodies (Southern Biotech) with a TMB solution (BioLegend). For anti-dsDNA and anti-ssDNA antibodies, plates precoated with dsDNA or ssDNA were added with 1:30 diluted serum samples and were similarly detected with HRP-conjugated secondary antibodies.

To detect antinuclear antibodies, HEp-2 cells adhered to slides were fixed and permeabilized and incubated with 1:40 diluted serum samples. Cells were stained with goat anti-mouse IgG (H+L)–FITC (1:5000 dilution, Southern Biotech) and DAPI (500 ng/ml, Thermo Fisher Scientific). Slides were imaged on a Leica SP5 confocal microscope.

### RNA-sequencing and transcriptomic analysis

Total RNA was isolated from CD4^+^Foxp3YFP^+^ Tregs and CD4^+^Foxp3YFP^-^ Tcons from WT*-Foxp3^YFPCre/YFPCre^* and *Dgka^-/-^z^fl/fl^-Foxp3^YFPCre/YFPCre^* mice (10- to 12-week-old) using the RNeasy Plus Mini kit (Qiagen). RNA-sequencing libraries were prepared using Truseq stranded mRNA kit (Illumina) according to the manufacturer’s protocol and sequenced at the Sequencing and Genomic Technologies Shared Resource at Duke University using the Illumina HiSeq2500 single-end 50 bp platform. Raw sequencing reads were processed with FASTQC to screen eligible samples, and then single-end reads were trimmed for adaptor sequences and filtered with the SoapNuke tool. Trimmed reads were aligned by STAR (2.5.1b) to the mouse Ensembl genome (Ensembl, GRCm38.p5) with Ensembl annotation (Mus_musculus.GRCm38.84.gtf). Counting of reads on annotated transcripts was performed with htseq-count (0.6.0). The limma (3.38.3) Bioconductor library has been used for counts normalization and differential analysis between the transcriptomes of the four WT and DKO Tregs. DEGs were subjected to enrichment analysis using ClusterProfiler (2.2.7). Biological processes in pathways in KEGG were chosen as significantly enriched terms with a p value less than 0.05.

### Statistical analysis

Statistical analyses were performed with Prism 5 (GraphPad). Each data point represents an individual mouse, and the number of data points correspoinds to the sample size (n). Error bars represent SEM. p Values were calculated using two-tailed paired or unpaired Student’s t test. p Values of less than 0.05 were considered significant. *p<0.05, **p<0.01, ***p<0.001, and ****p<0.0001 (Student’s t test).

### Study approval

All animal experiments were performed according to protocols approved by the IACUC of Duke University.

## Data Availability

RNA sequencing data has been deposited in the GEO database (GSE276410). The following dataset was generated: LiL ZhongX 2025Synergistic Role of DGKalpha and zeta in Regulatory T cell function, stability, and pathogenecityNCBI Gene Expression OmnibusGSE276410

## Additional files

Supplementary file 1. Differentially expressed genes between WT and αζDKO Tregs.

Supplementary file 2. KEGG pathway analysis of WT and αζDKO Tregs.

Supplementary file 3. Differentially expressed genes between CD4^+^Foxp3YFP^-^ T cells from *Dgka^-/-^z^f/f^-Foxp3^YFPCre/YFPCre^* and *WT-Foxp3^YFPCre/YFPCre^* mice.

Supplementary file 4. Enriched KEGG pathways in CD4^+^Foxp3YFP^-^ T cells from *Dgka^-/-^z^f/f^-Foxp3^YFPCre/YFPCre^* and *WT-Foxp3^YFPCre/YFPCre^* mice.

Supplementary file 5. Genes differentially expressed in both αζDKO Tregs and Tcons.

Supplementary file 6. List of key reagents.

MDAR checklist
