# Supplementary material for: DGKα and ζ deficiency causes regulatory T-cell dysregulation, destabilization, and conversion to pathogenic T-follicular helper cells to trigger IgG1-predominant autoimmunity
Source: eLife. 2026 Sep 18;14:RP105212. doi: 10.7554/eLife.105212 (PMC13588621; doi:10.7554/eLife.105212)
Supplement: Peer review history

# Editor report: eLife Assessment

**DOI:** 10.7554/eLife.105212.3.sa0

**Authors:** Ana Maria Faria

**Affiliations:** Universidade Federal de Minas Gerais Brazil

**Keywords:** Important, Solid

This study presents an **important** finding on the signaling mechanisms underlying Treg cell homeostasis by identifying the simultaneous requirement of diacylglycerol (DAG) kinases (DGK) alpha and zeta for Foxp3+ Treg cell function and follicular responses, with implications for the pathogenesis of some autoimmune diseases. Whereas data based on the characterization of double knock-out mice (for DGK alpha and zeta) is **solid**, showing the emergence of autoimmune manifestations, the study has gaps in its experimental approaches since it is not clear what can be attributed to the simultaneous DKGα and ζ deficiency, versus the individual deficiency of either one. Experiments on the pathogenic potential of the DKO Tregs in the absence of other T-cells were not presented and results on the role of CD25 downregulation and CD28-independent activation of Treg cells were not properly discussed. Nonetheless, the reported data would be of interest to immunologists working on T-cell intracellular signaling and autoimmunity.

# Referee report: Reviewer #1 (Public review)

**DOI:** 10.7554/eLife.105212.3.sa1

Summary:

The manuscript by Li and colleagues describes the impact of deficiency on the DKGα and ζ on Treg cells and follicular responses. The experimental approach is based on the characterization of double KO mice that show the emergence of autoimmune manifestations that include the production of autoantibodies. Additionally, there is an increase in Tfh cells, but also Tfr cells in these mice deficient in both DKGα and ζ. Although the observations are interesting, the interpretation of the observations is difficult in the absence of data related to single mutations. While a supplementary figure shows that the autoimmune manifestations are more severe in the DKGα and ζ deficient mice, prior observations show that a single DKGα deficiency has an impact on Treg homeostasis. As such, the contribution of the two chains to the overall phenotype is hard to establish.

Strengths:

Well-conducted experiments with informative mouse models with defined genetic defects.

Weaknesses:

The major weakness is the lack of clarity concerning what can be attributed to simultaneous DKGα and ζ deficiency versus deficiency on DKGα or ζ alone. Technical concerns related to a number of figures were raised in the initial report and not adequately addressed by the authors in the revised manuscript.

In conclusion, the claims in the manuscript are not convincingly supported by the data,

# Referee report: Reviewer #2 (Public review)

**DOI:** 10.7554/eLife.105212.3.sa2

Summary:

In this manuscript, Li et al investigates the combined role of diacylglycerol (DAG) kinases (DGK) a and z in Foxp3+ Treg cells function that prevent autoimmunity. The authors generated DGK a and z Treg-specific double knock out mice (DKO) by crossing Dgkalpha-/- mice to DgKzf/f and Foxp3YFPCre/+ mice. The resulting "DKO" mice thus lack DGK a in all cells and DGK and z in Foxp3+Treg cells. The authors show that the DKO mice spontaneously develop autoimmunity, characterized by multiorgan inflammatory infiltration and elevated anti double strand DNA (dsDNA), -single strand DNA (ssDNA), and -nuclear autoantibodies. The authors attribute the DKO mice phenotype to Foxp3+Treg dysfunction, including accelerated conversion into "exTreg" cells with pathogenic activity. Interestingly, the combined deficiency of DGK a and z seems to release Treg cell dependence on CD28-mediated costimulatory signals, which the authors show by crossing their DKO mice to CD28-/- mice (TKO mice), which also develop autoimmunity.

Strengths:

The phenotypes of the mutant mice described in the manuscript are striking, and the authors provide a comprehensive analysis of the functional processes alters by the lack of DGKs.

Weaknesses:

One aspect that could be better explored is the direct role of "ex-Tregs" in causing pathogenesis in the models utilized.

But overall, this is an important report that makes a significant addition to the understanding of DAG kinases to Treg cells biology.

# Author comment: Author response

**DOI:** 10.7554/eLife.105212.3.sa3

**Authors:** Lei Li, Hongxiang Huang, Hongxia Wang, Yun Pan, Huishan Tao, Shimeng Zhang, Peer Karmaus, Michael B Fessler, John Sleasman, Xiao-Ping Zhong

**Affiliations:** Duke Medical Center Durham United States; Duke University Medical Center Durham United States; Duke Medical Center Durham United States; Duke Medical Center Durham United States; Duke Medical Center Durham United States; Duke Medical Center Durham United States; National Institute of Environmental Health Sciences Research Triangle Park United States; National Institute of Environmental Health Sciences Research Triangle Park United States; Duke Medical Center Durham United States; Duke Medical Center Durham United States

The following is the authors’ response to the original reviews

**Public Reviews:**

**Reviewer #1 (Public review):**

Summary:

The manuscript by Li and colleagues describes the impact of deficiency on the DKGα and ζ on Treg cells and follicular responses. The experimental approach is based on the characterization of double KO mice that show the emergence of autoimmune manifestations that include the production of autoantibodies. Additionally, there is an increase in Tfh cells, but also Tfr cells in these mice deficient in both DKGα and ζ. Although the observations are interesting, the interpretation of the observations is difficult in the absence of data related to single mutations. While a supplementary figure shows that the autoimmune manifestations are more severe in the DKGα and ζ deficient mice, prior observations show that a single DKGα deficiency has an impact on Treg homeostasis. As such, the contribution of the two chains to the overall phenotype is hard to establish.

Strengths:

Well-conducted experiments with informative mouse models with defined genetic defects.

Weaknesses:

The major weakness is the lack of clarity concerning what can be attributed to simultaneous DKGα and ζ deficiency versus deficiency on DKGα or ζ alone.

Some interpretations are also not conclusively supported by data.

We appreciate the reviewer 1’s positive comments about our manuscript and for the suggestion to include DGKα‑ or DGKζ‑single‑knockout (SKO) Tregs for the mechanistical studies. Unfortunately, performing this sound simple but truly extensive experiment would exceed our current budget and personnel capacity. Importantly, it is well known that DGKα and DGKζ act redundantly or synergistically in T cells, with single loss producing minimal or partial phenotypes compared with the double knockout. The comprehensive mechanistic data already presented for DGKαζ‑DKO Tregs therefore capture the combined functional and mechanistical deficit that is most relevant to DGK functions in Treg biology, and they support the conclusions drawn in this manuscript. The reviewer also pointed out some interpretation issues such as CD25 down regulation in Tfr cells and some minor issues. We appreciate the reviewer’s expertise and have revised the text and discussion accordingly.

**Reviewer #2 (Public review):**

Summary:

In this manuscript, Li et al investigate the combined role of diacylglycerol (DAG) kinases (DGK) α and ζ in Foxp3+ Treg cells function that prevent autoimmunity. The authors generated DGK α and ζ Treg-specific double knockout mice (DKO) by crossing Dgkalpha-/- mice to DgKzf and Foxp3YFPCre/+ mice. The resulting "DKO" mice thus lack DGK α in all cells and DGK ζ in Foxp3+Treg cells. The authors show that the DKO mice spontaneously develop autoimmunity, characterized by multiorgan inflammatory infiltration and elevated anti-double-strand DNA (dsDNA), -single-strand DNA (ssDNA), and -nuclear autoantibodies. The authors attribute the DKO mice phenotype to Foxp3+Treg dysfunction, including accelerated conversion into "exTreg" cells with pathogenic activity. Interestingly, the combined deficiency of DGK α and ζ seems to release Treg cell dependence on CD28-mediated costimulatory signals, which the authors show by crossing their DKO mice to CD28-/- mice (TKO mice), which also develop autoimmunity.

Strengths:

The phenotypes of the mutant mice described in the manuscript are striking, and the authors provide a comprehensive analysis of the functional processes altered by the lack of DGKs.

Weaknesses:

One aspect that could be better explored is the direct role of "ex-Tregs" in causing pathogenesis in the models utilized.

However, overall, this is an important report that makes a significant addition to the understanding of DAG kinases in Treg cell biology.

We greatly appreciate reviewer 2’s positive comments about the manuscript. The data we presented in the manuscript show that DGKαζDKO Tregs but not WT Tregs are able to trigger autoimmunity in T cell deficient mice in the presence of WT CD4 T cells support that DGKαζDKO Tregs are pathogenic. Reviewer 2 suggested to test the direct role of DGKαζDKO Treg/ex-Tregs in the pathogenesis of autoimmune diseases in the absence of conventional T cells. This is really an interesting idea that we will test it in the future should recourse for executing the experiment become available.
